# Systems pharmacology dissection of *Epimedium* targeting tumor microenvironment to enhance cytotoxic T lymphocyte responses in lung cancer

**DOI:** 10.18632/aging.202410

**Published:** 2021-01-17

**Authors:** Chao Huang, Zhihua Li, Jinglin Zhu, Xuetong Chen, Yuanyuan Hao, Ruijie Yang, Ruifei Huang, Jun Zhou, Zhenzhong Wang, Wei Xiao, Chunli Zheng, Yonghua Wang

**Affiliations:** 1 Bioinformatics Center, College of Life Sciences, Northwest A&F University, Yangling 712100, Shaanxi, China; 2 Key Laboratory of Resource Biology and Biotechnology in Western China, Ministry of Education, School of Life Sciences, Northwest University, Xi’an 710069, China; 3 State Key Laboratory of New-Tech for Chinese Medicine Pharmaceutical Process, Jiangsu Kanion Pharmaceutical, Co., Ltd., Lianyungang 222001, China

**Keywords:** systems pharmacology, NSCLC, tumor microenvironment, epimedium, icaritin

## Abstract

The clinical notably success of immunotherapy fosters an enthusiasm in developing drugs by enhancing antitumor immunity in the tumor microenvironment (TME). *Epimedium*, is a promising herbal medicine for tumor immunotherapy due to the pharmacological actions in immunological function modulation and antitumor. Here, we developed a novel systems pharmacology strategy to explore the polypharmacology mechanism of *Epimedium* involving in targeting TME of non-small cell lung cancer (NSCLC). This strategy integrates the active compounds screening, target predicting, network pharmacology analysis and onco-immune interacting to predict the potential active compounds that trigger the antitumor immunity. Icaritin (ICT), a major active ingredient of *Epimedium*, was predicted to have good drug-like properties and target immune microenvironment in NSCLC via regulating multiple targets and pathways. Then, we evidenced that the ICT effectively inhibited tumor growth in LLC tumor-bearing mice and increases the infiltration of CD8+ T cells in TME. In addition, we demonstrated that ICT promotes infiltration of CD8^+^ T cells in TME by downregulating the immunosuppressive cytokine (TNF-α, IL10, IL6) and upregulating chemotaxis (CXCL9 and CXCL10). Overall, the systems pharmacology strategy offers an important paradigm to understand the mechanism of polypharmacology of natural products targeting TME.

## INTRODUCTION

Lung cancer is the leading cause of cancer-related mortality worldwide, and non-small-cell lung cancer (NSCLC) represents the major histological subtype of the disease [1]. In recent years, studies have shown that triggering the antitumor immunity of CD8^+^ T cells in the tumor microenvironment (TME) by immunotherapy brings prominent and long-lasting clinical benefits for NSCLC [2]. However, only a small number of patients could benefit from such approaches alone [3]. The possible explanation is that TME is a complex ecosystem consisting of various cells and its formation and dynamic changes are regulated by multiple targets, multiple signaling pathways and multiple cells [4]. The tumor microenvironment (TME) is composed not only of tumor cells but also of stromal cells, inflammatory cells, vasculature, and extracellular matrices (ECM). Tumor cells escape immunosurveillance by increasing signaling through coinhibitory receptors of T cells or reducing the availability of antigen for presentation. In the TME, inflammatory cells secrete chemokines and cytokines to promote the growth and metastasis of tumor cells, genetic mutations, and angiogenesis [5]. The tumor vasculature excludes of effector lymphocytes from the tumor microenvironment by physical means. ECM enables cancer cells to subvert the immune component of the microenvironment via providing both a biochemical and biomechanical context. Stromal cells express numerous surface and secreted molecules such as IL-6, NO, PGE2 and TGFβ which directly suppress CD4^+^ and CD8^+^T cells and activate immunosuppressive myeloid cells. For this reason, combination strategy toward multi-target anticancer therapy is a promising field in development of immunotherapeutic drug to overcome this challenge [6].

Accumulated studies have shown that natural products can exert anti-tumor effects by regulating immune-related signaling pathways and targets [7–11]. At the same time, natural products have multi-target and multi-pathway characteristics compared with conventional therapeutic drugs, which can play a key role in the regulation of complex networks in TME [12–15]. Thus, natural products that promote these effects via a wide variety of mechanisms are gradually becoming a huge resource pool for the development of antitumor drugs [16–18]. For example, *Epimedium,* a famous herbal medicine, is widely used as a tonic, aphrodisiac and antirheumatic in China, Japan and Korea for more than 2000 years. Studies and clinical practices have demonstrated that *Epimedium* is a relatively non-toxic nutritional herbal product and has wide pharmacological actions, especially in immunological function modulation, anti-tumor [19–21]. Hitherto, more than 100 compounds have been isolated from *Epimedium*. However, it is unclear that which compound determine the pharmacological effect, and the mechanism(s) of how *Epimedium* promotes the antitumor immunity in NSCLC remains enigmatic.

**Figure 1:**
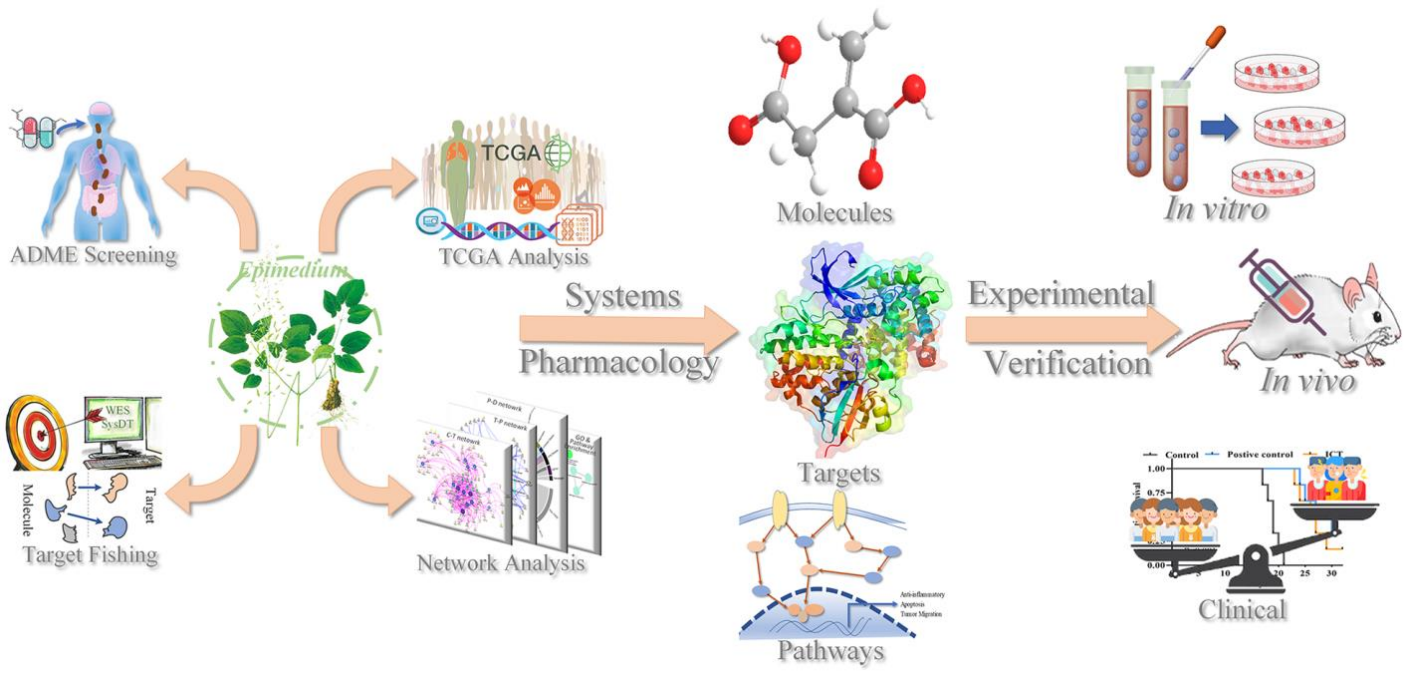
**The workflow of the systems pharmacology method.**

In this work, we used a systems pharmacology approach to elucidate the action mechanism of polypharmacology molecules of *Epimedium* targeting TME for the treatment of NSCLC. We screened the polypharmacology molecules of *Epimedium*, predicted the targets of active compounds, constructed the networks, and linked the targets to the immune phenotype in lung cancer patients. These results indicated that polypharmacology molecules of *Epimedium* targeted several kinds of tumor-related factors and signaling pathways in the TME, such as inflammation, apoptosis, and migration. Additionally, we used *in vivo* and *in vitro* experiments to verify the antitumor efficacy of icaritin (ICT), since it is a major active ingredients of *Epimedium* and was predicted to have a good drug-like and pharmacological properties in targeting the NSCLC immune microenvironment. The LLC tumor-bearing mice model evidenced that tumor volume was significantly reduced and survival time was significantly extended after treatment with ICT. Furthermore, transcriptome sequencing and immunofluorescence showed that ICT could target the TME to upregulate the immune signaling pathway and T cell chemokines, thereby increasing the infiltration of CD8+ T cells to achieve antitumor immunity. In conclusion, systems pharmacology provided an important reference to insight into the action mechanism of polypharmacology molecules of *Epimedium* targeting TME against NSCLC, and offered a significant strategy for development and application of natural products for immunotherapy. As Figure 1 showed the process flow framework of this study.

## RESULTS

### Polypharmacology molecules of *Epimedium* for the treatment of NSCLC

To elucidate the polypharmacology molecules of *Epimedium* targeting TME on NSCLC, we firstly achieved the compounds in *Epimedium* by searching literatures and Traditional Chinese Medicine Systems Pharmacology Database (TCMSP), which results in 130 compounds. Then, 16 ingredients of *Epimedium* with favorable pharmacokinetic characteristics were determined by ADME (Absorption, Distribution, Metabolism, and Excretion) screening conditions satisfied as previous study [22], i.e., oral bioavailability (OB) ≥ 30%, half-life (HL) ≥ 4 and drug-likeness (DL) ≥ 0.18 (Table 1). Among them, flavonoid plays an important role in the treatment and prevention of various cancers. For instance, ICT (MOL056 OB=45.41%, DL=0.44, HL=15.01) has various beneficial activities which inhibit tumor growth or target the TME [23–25]. Kaempferol (MOL044 OB=41.88%, DL=0.24, HL=14.74) induces cell cycle arrest in G1 and G2/M by inhibiting the activity of CDK2, CDK4 and Cdc2, leading to apoptosis [26]. Moreover, sitosterol (MOL042 OB=36.91%, DL=0.75, HL=5.37) has been shown antimigration in various lung cancer cell types [27–29]. Linoleate acetate (MOL008 OB = 42.10%, DL = 0.20, HL = 7.48) is used in the treatment of NSCLC to inhibit inflammatory factors COX, LOX and TNF-α in the TME, which were closely related to their immune activities [30, 31].

**Table 1:** Chemical information and network parameters of 16 active compounds from *Epimedium*.

**MOL-ID**	**Compounds**	**Structure**	**Categories**	**OB**	**DL**	**HL**	**Degree**
MOL056	Icaritin	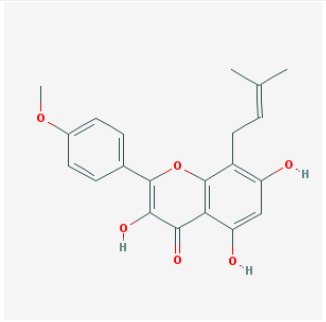	Flavonoids	45.41	0.44	15.01	32
MOL044	Kaempferol	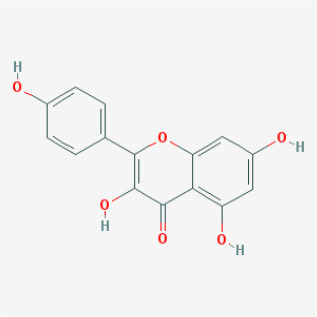	Flavonoids	41.88	0.24	14.74	31
MOL033	Chrysoeriol	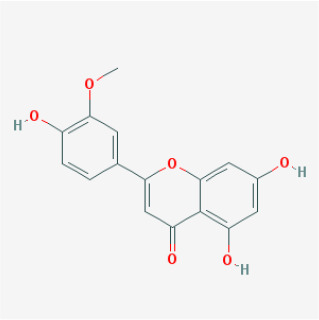	Flavonoids	35.85	0.27	16.31	28
MOL039	8-Isopentenyl-kaempferol	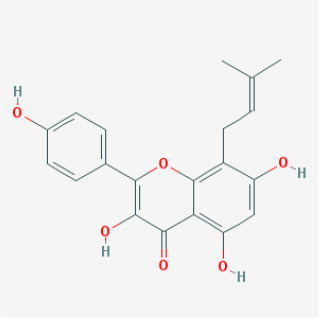	Flavonoids	38.04	0.39	15.37	24
MOL067	Yinyanghuo C	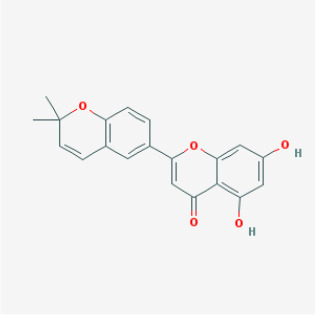	Flavonoids	45.67	0.50	15.74	24
MOL069	Yinyanghuo E	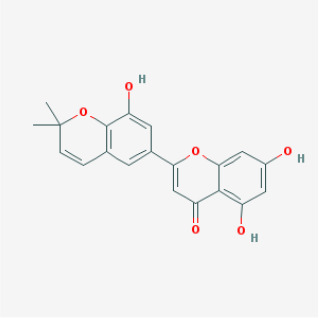	Flavonoids	51.63	0.55	15.47	22
MOL074	8-(3-methylbut-2-enyl)-2-phenyl-chromone	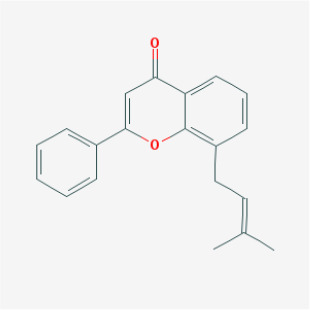	Flavonoids	48.54	0.25	18.73	20
MOL012	(2S)-7-hydroxy-2-(4-hydroxyphenyl)-4-chromanone	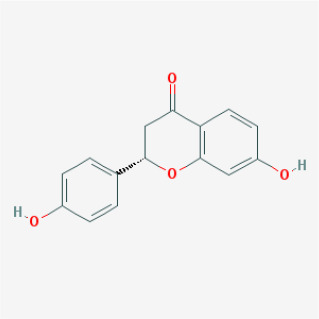	Flavonoids	32.76	0.18	17.89	19
MOL065	Yinyanghuo A	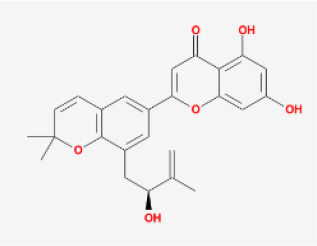	Flavonoids	56.96	0.77	14.44	13
MOL106	Icariin	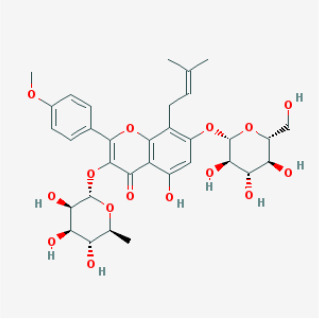	Flavonoids	41.58	0.61	19.93	11
MOL004	24-epicampesterol	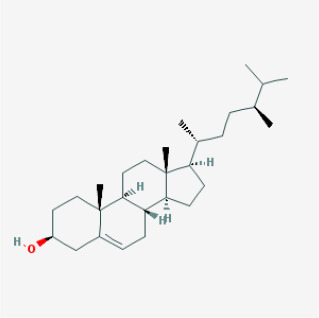	Sterols	37.58	0.71	4.50	18
MOL010	Poriferast-5-en-3beta-ol	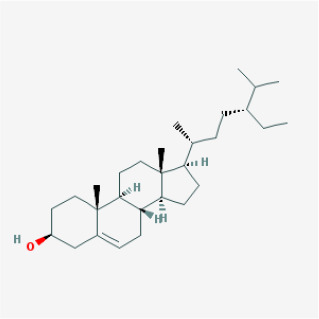	Sterols	36.91	0.75	5.07	17
MOL042	Sitosterol	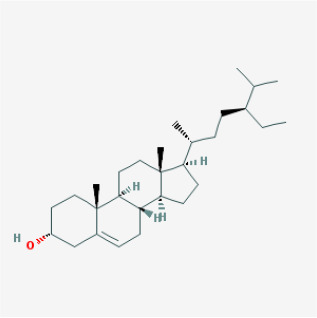	Sterols	36.91	0.75	5.37	17
MOL008	Linoleyl acetate	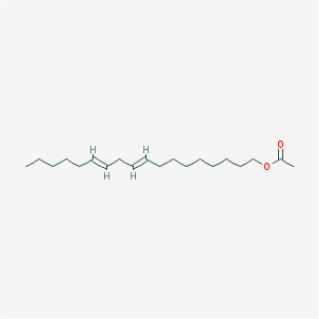	Lignans	42.10	0.20	7.48	18
MOL063	C-Homoerythrinan, 1,6-didehydro 3,15,16-trimethoxy-, (3.beta.)-	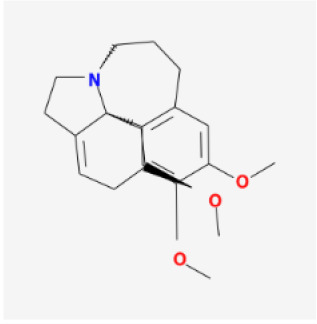	Others	39.14	0.49	6.58	18
MOL077	Anhydroicaritin-3-O-alpha-L-rhamnoside	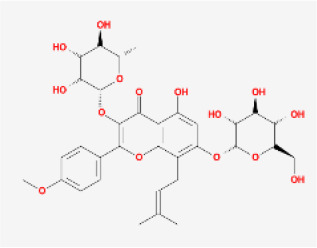	Others	41.58	0.61	16.23	12

OB, oral bioavailability; DL, drug-likeness; HL, Half-life.

### Polypharmacology mechanism of *Epimedium* targeting TME to treat NSCLC

To clarify the potential action mechanism of the multi-target of candidate compounds targeting TME for the treatment of NSCLC. 389 candidate targets for the 16 potential active compounds were obtained by utilizing the similarity ensemble approach (SEA), weighted ensemble similarity (WES) and systematic drug targeting tool (SysDT) methods. After screening, we retrieved 89 potential targets (Table 2 and Supplementary Tables 1, 2). ESR1 and AR are the morphogenetic factors that regulate cell proliferation, differentiation, angiogenesis, and the development of some tumors [32, 33], which were predicted to link the *Epimedium* to cancer with highest scores. In line with our prediction, studies show that ICT can target ESR1 and AR to control the growth of advanced breast and prostate cancer [24, 34], which confirms the predictive accuracy of our approach. Except for ESR1 and AR, we also predicted many targets related to immune and inflammation with high scores, such as PPARG, PTGS2, PTGS1, ALOX5. In the subsequent study, we mainly focused on these targets. Related studies have shown that regulating the expression of these targets could promote apoptosis and inhibit inflammation in the TME. For example, the COX2 has a variety of biological activities such as inhibiting cell apoptosis, promoting cell proliferation, inhibiting immune surveillance and promoting angiogenesis in the TME, thus playing a key role in the occurrence and development of tumors [35–37]. Therefore, we described the relationship between targets and NSCLC from the following three aspects.

**Table 2:** The targets information of *Epimedium.*

**UniProt-ID**	**Protein names**	**Gene names**	**Degree**	**Species**
P03372	Estrogen receptor	ESR1	14	homo sapiens
P10275	Androgen receptor	AR	14	homo sapiens
Q92731	Progesterone receptor	ESR2	12	homo sapiens
P37231	Peroxisome proliferator activated receptor gamma	PPARG	11	homo sapiens
P35354	Prostaglandin G/H synthase 2	PTGS2	11	homo sapiens
Q9UNQ0	ATP-binding cassette sub-family G member 2	ABCG2	10	homo sapiens
P49841	Glycogen synthase kinase-3 beta	GSK3B	10	homo sapiens
Q16678	Cytochrome P450 1B1	CYP1B1	10	homo sapiens
P23219	Prostaglandin G/H synthase 1	PTGS1	9	homo sapiens
P11309	Proto-oncogene serine/threonine-protein kinase Pim-1	PIM1	9	homo sapiens
P35228	Nitric oxide synthase, inducible	NOS2	8	homo sapiens
Q16539	Mitogen-activated protein kinase 14	MAPK14	8	homo sapiens
P24941	Cell division protein kinase 2	CDK2	8	homo sapiens
P47989	Xanthine dehydrogenase/oxidase	XDH	8	homo sapiens
P16220	Cyclic AMP-responsive element-binding protein 1, CREB-1, cAMP-responsive element-binding protein 1	CREB1	7	homo sapiens
P53355	Death-associated protein kinase 1	DAPK1	7	homo sapiens
P60568	Interleukin-2	IL2	7	homo sapiens
P51812	Death-associated protein kinase 1	RPS6KA3	7	homo sapiens
P08183	Multidrug resistance protein 1	ABCB1	7	homo sapiens
P15692	Vascular endothelial growth factor A	VEGFA	6	homo sapiens
P15121	Aldo-keto reductase family 1 member B1	AKR1B1	6	homo sapiens
P33527	ATP-binding cassette sub-family C member 1	ABCC1	6	homo sapiens
P09917	Arachidonate 5-lipoxygenase	ALOX5	5	homo sapiens
P06401	Progesterone receptor	PGR	4	homo sapiens
P00918	Carbonic anhydrase 2	CA2	4	homo sapiens
P11511	Aromatase	CYP19A1	4	homo sapiens
P11473	Vitamin D3 receptor	VDR	4	homo sapiens
P29474	Nitric-oxide synthase, endothelial	NOS3	4	homo sapiens
P07550	Beta-2 adrenergic receptor	ADRB2	4	homo sapiens
P02766	Transthyretin	TTR	4	homo sapiens
Q12809	Potassium voltage-gated channel subfamily H member 2	KCNH2	4	homo sapiens
Q08828	Adenylate cyclase type 1	ADCY1	3	homo sapiens
P00915	Carbonic anhydrase 1	CA1	3	homo sapiens
Q07973	1,25-dihydroxyvitamin D (3) 24-hydroxylase, mitochondrial	CYP24A1	3	homo sapiens
O15439	Multidrug resistance-associated protein 4	ABCC4	3	homo sapiens
P51449	Nuclear receptor ROR-gamma	RORC	3	homo sapiens
Q12772	Sterol regulatory element-binding protein 2	SREBF2	3	homo sapiens
P02774	Vitamin D-binding protein	GC	3	homo sapiens
P48736	Phosphatidylinositol-4,5-bisphosphate 3-kinase catalytic subunit, gamma isoform	PIK3CG	3	homo sapiens
P04798	Cytochrome P450 1A1	CYP1A1	3	homo sapiens
O76074	cGMP-specific 3',5'-cyclic phosphodiesterase	PDE5A	3	homo sapiens
P08709	Coagulation factor VII	F7	3	homo sapiens
Q9UBM7	7-dehydrocholesterol reductase	DHCR7	2	homo sapiens
P04150	Glucocorticoid receptor	NR3C1	2	homo sapiens
P07451	Carbonic anhydrase 3	CA3	2	homo sapiens
Q09472	Histone acetyltransferase p300	EP300	2	homo sapiens
Q12791	Calcium-activated potassium channel subunit alpha-1	KCNMA1	2	homo sapiens
P05091	Aldehyde dehydrogenase, mitochondrial	ALDH2	2	homo sapiens
Q04206	Transcription factor p65	RELA	2	homo sapiens
P27695	DNA - (apurinic or apyrimidinic site) lyase	APEX1	2	homo sapiens
Q9UHC3	Acid-sensing ion channel 3	ASIC3	2	homo sapiens
P09382	Galectin-1	LGALS3	2	homo sapiens
P14679	Tyrosinase	TYR	2	homo sapiens
P05230	Fibroblast growth factor 1	FGF1	1	homo sapiens
P21554	Cannabinoid receptor 1	CNR1	1	homo sapiens
P34972	Cannabinoid receptor 2	CNR2	1	homo sapiens
Q13822	Ectonucleotide pyrophosphatase/phosphodiesterase family member 2	ENPP2	1	homo sapiens
P23141	Liver carboxylesterase 1	CES1	1	homo sapiens
P15090	Fatty acid-binding protein, adipocyte	FABP4	1	homo sapiens
P17252	Protein kinase C alpha type, PKC-A, PKC-alpha	PRKCA	1	homo sapiens
P47712	Cytosolic phospholipase A2, cPLA2	PLA2G4A	1	homo sapiens
Q53EL6	Programmed cell death protein 4	PDCD4	1	homo sapiens
O95136	Sphingosine 1-phosphate receptor 2, S1P receptor 2, S1P2	S1PR2	1	homo sapiens
P08842	Steryl-sulfatase	STS	1	homo sapiens
O60603	Toll-like receptor 2	TLR2	1	homo sapiens
Q8NER1	Transient receptor potential cation channel subfamily V member 1	TRPV1	1	homo sapiens
P14780	Matrix metalloproteinase-9	MMP9	1	homo sapiens
P05412	Transcription factor AP-1	JUN	1	homo sapiens
P00338	L-lactate dehydrogenase A chain, LDH-A	LDHA	1	homo sapiens
Q9UDY8	Mucosa-associated lymphoid tissue lymphoma translocation protein 1	MALT1	1	homo sapiens
Q16790	Carbonic anhydrase 9	CA9	1	homo sapiens
P05177	Cytochrome P450 1A2	CYP1A2	1	homo sapiens
P28702	Retinoic acid receptor RXR-beta	RXRB	1	homo sapiens
P35367	Histamine H1 receptor	HRH1	1	homo sapiens
Q15788	Nuclear receptor coactivator 1	NCOA1	1	homo sapiens
P15559	NAD(P)H dehydrogenase [quinone] 1	NQO1	1	homo sapiens
P22460	Potassium voltage-gated channel subfamily A member 5	KCNA5	1	homo sapiens
P08913	Alpha-2A adrenergic receptor	ADRA2A	1	homo sapiens
P08908	5-hydroxytryptamine receptor 1A, 5-HT-1A, 5-HT1A	HTR1A	1	homo sapiens
P18089	Alpha-2B adrenergic receptor	ADRA2B	1	homo sapiens
Q15822	Neuronal acetylcholine receptor subunit alpha-2	CHRNA2	1	homo sapiens
P14416	D (2) dopamine receptor	DRD2	1	homo sapiens
P36544	Neuronal acetylcholine receptor subunit alpha-7	CHRNA7	1	homo sapiens
P30532	Neuronal acetylcholine receptor subunit alpha-5	CHRNA5	1	homo sapiens
Q9Y271	Cysteinyl leukotriene receptor 1	CYSLTR1	1	homo sapiens
O43570	Carbonic anhydrase 12	CA12	1	homo sapiens
P78527	DNA-dependent protein kinase catalytic subunit, DNA-PK catalytic subunit, DNA-PKcs	PRKDC	1	homo sapiens
P02787	Serotransferrin, Transferrin	TF	1	homo sapiens
Q9NYY3	Serine/threonine-protein kinase PLK2	PLK2	1	homo sapiens

**Figure 2:**
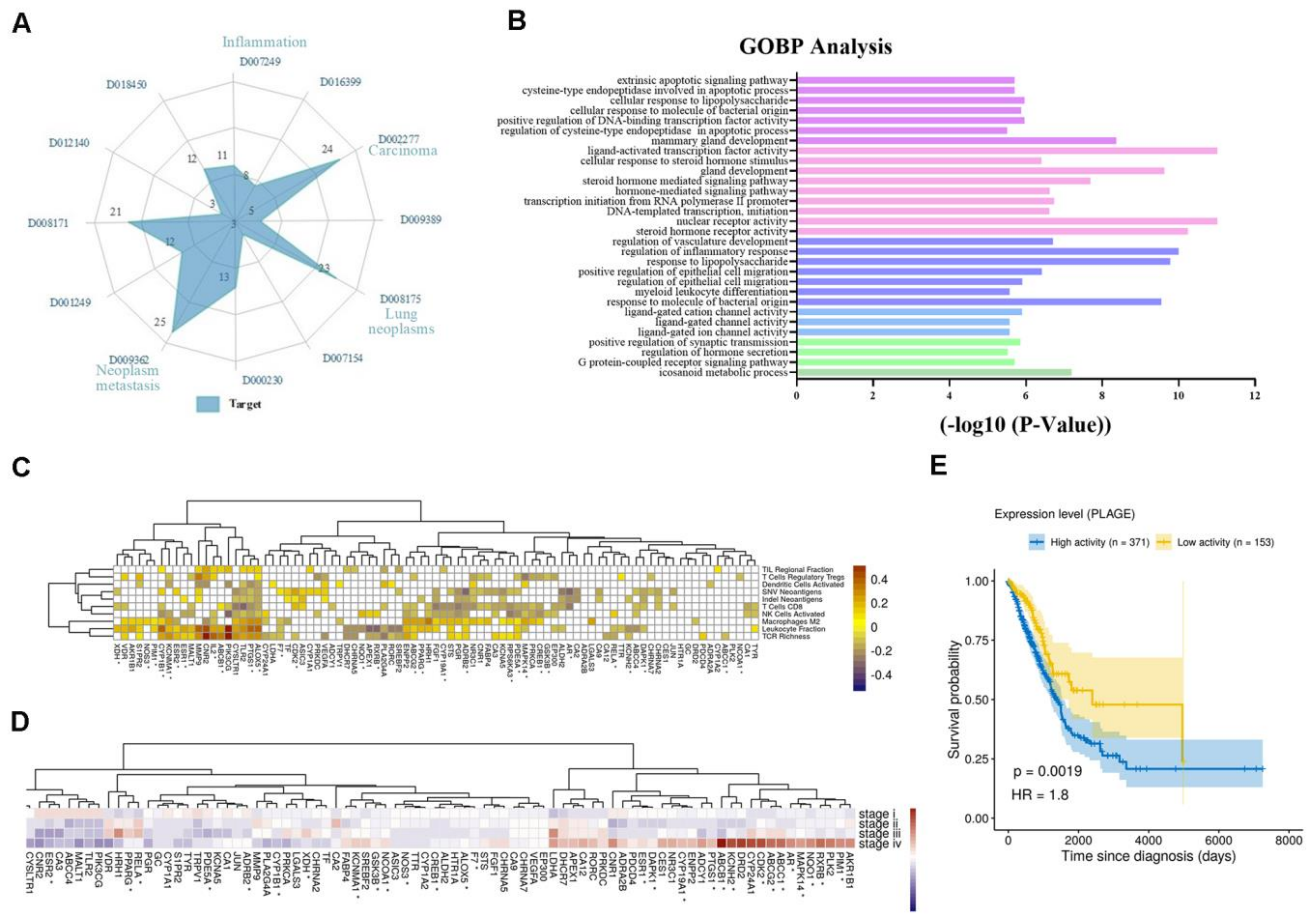
**Analysis of multi-target of polypharmacology molecules of *Epimedium* treatment of NSCLC.** (**A**) In an equi-angular spokes radar chart, each spoke represents a class of diseases, and the length of the spokes is the distribution data, which is proportional to the quantity of target proteins relative to the homologous disease. (**B**) The y-axis shows significantly enriched ‘Biological Process’ categories in GO of the target genes, and the x-axis shows the enrichment scores of these terms *(P*-value < 0.05). A specific color represents a class of biological processes. (**C**) The heatmap of Pearson correlation coefficients (PCCs) between gene expression level of *Epimedium* targets and immune phenotypes. The Benjamini-Hochberg (BH) adjusted p-values of PCCs <0.05 were shown as white color. Note that the genes labeled with an asterisk represents the targets for ICT. (**D**) Average expression of *Epimedium* targets among clinical staging (stages I–IV) in TCGA LUDA patients. For each gene, the expression was normalized by z-score transformation. Note that the genes labeled with an asterisk represents the targets for ICT. (**E**) Prognostic value of the *Epimedium* targets for overall survival of human LUAD patients comparing high and low activity. X-axis shows time for survival (days) and y-axis shows the probability of survival, where 1.0 corresponds to 100 percent.

Firstly, we analyzed the direct or indirect relationships between targets and diseases, as shown in Figure 2A. The radar chart analysis showed that the targets we screened were closely related to carcinoma (D002277), neoplasm metastasis (D009362), lung neoplasms (D008175), inflammation (D007249) and so on, and these targets simultaneously targeted two or more diseases. Moreover, these diseases were also affected by multiple targets simultaneously. For instance, lung neoplasms (D008175) were regulated by 23 targets such as iNOS, Bcl2 and PKC that have anti-inflammatory, pro-apoptotic effects in the TME. Then, we performed the Gene Ontology (GO) biological processes enrichment for these targets by using ClueGO and visualized them by Enrichment Map with the threshold of *P*-value ≤ 0.05 (Supplementary Table 3). We found that most of these targets were strongly associated with various biological processes in the TME, such as nuclear receptor activity, response to lipopolysaccharide, transcription initiation from RNA polymerase II promoter and so on (Supplementary Figure 1). Among these groups, regulation of inflammatory response, regulation of vasculature development and extrinsic apoptotic signaling pathway were all closely associated with NSCLC (Figure 2B). For example, by down-regulating angiogenic factors, the activation of vascular endothelial cells was inhibited, and the proliferation and migration of endothelial cells were prevented, thereby achieving the effect of treating tumors.

In addition, we further analyzed the correlation between gene expression of *Epimedium* targets (rows) and scores of ten anti-tumor immunity related phenotypes (columns) within 533 LUDA patients in TCGA. The phenotypes include (i) the cell abundances of 6 major immune cell types (leukocyte, T cells CD8, activated NK cells, activated dendritic cells, Tregs, macrophages M2), which were deconvoluted from tumor transcriptome, (ii) the neoantigen number predicted from SNVs and Indels, (iii) TCR richness inferenced from tumor RNA-Seq and (iv) the TIL regional fraction estimated from the tissue image. These immune phenotypes were identified based on the methods established by llya Shmulevich et al. [38]. And the degree of association between targets and immune phenotypes is reflected by Pearson correlation coefficient. As shown in Figure 2C, we correlated targets with immune phenotypes, resulting in 315 significant associations (Benjamini–Hochberg adjusted p-values <0.05) that involved 82 of 89 targets. Leukocyte fraction correlated with CNR2, PI3KCG, TLR2, PTGS1, ALOX5 and so on. Among them, inactivation of CNR2 or activation of ALOX5 has been reported to enhance leukocyte migration [39]. T cells regulatory (Tregs) correlated with in PTGS1, ALOX5, CNR2 and others, which could be immune-evading mechanisms. T cells CD8 correlated with many targets, as well as PGR, XDH, MALT1, and ADCY1, which may relate to CD8+ T infiltration. Additionally, we observed that the expression of these targets is related to clinical progression as clinical staging correlated with increased/decreased target expression (Figure 2D). Further, we evaluated the overall expression levels of the target set among the LUAD patients using the PLAGE (Pathway Level Analysis of Gene Expression) approach. We observed that higher expression of these target genes in tumor samples was negatively associated with patient survival in LUAD (HR = 1.8, log-rank test P-value = 0.0019) (Figure 2E).

**Figure 3:**
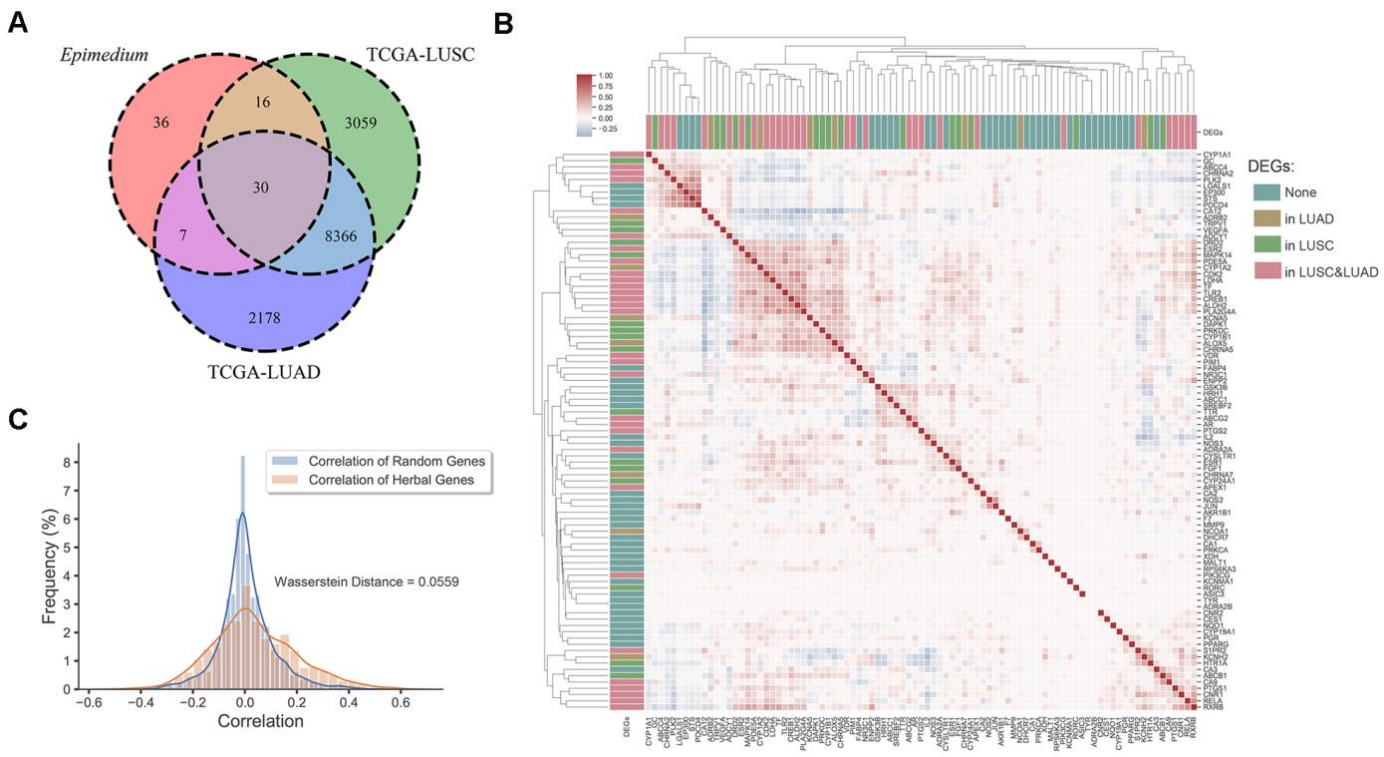
**Differentially expression gene analysis.** (**A**) Venn diagram of overlap genes in potential targets of *Epimedium* active ingredients and DEGs of LUSC and LUAD. Each region represents the number of genes. (**B**) Heatmap of the pairwise correlation of 89 potential genes of *Epimedium* active components. (**C**) Distribution diagram of random intergenic correlations and *Epimedium* intergenic correlation. Yellow indicates the correlation between the genes of *Epimedium*, and blue indicates the correlation between random genes (LUSC and LUAD).

Finally, analysis of differentially expressed genes data from the Genomic Data Commons (GDC) Data Portal [40] indicated that most potential targets of *Epimedium* overlapped with differentially expressed genes (DEGs) of lung adenocarcinoma (LUAD) and lung squamous cell carcinoma (LUSC). As shown in Figure 3A, we found that 51.7% of the targets overlapped with the DEGs of LUSC, 41.6% overlapped with the DEGs of LUAD, and 33.7% overlapped with the DEGs of LUAD and LUSC, respectively. In addition, we built an expression heatmap of 89 potential targets of *Epimedium* active components and based on the correlation analysis of the expression matrix, we found that the degree of association among overlapping genes in the Venn diagram was significantly higher, such as DAPK1, MAPK14, CDK2, LDHA, CYP1A2, etc. These well-correlated targets are closely related to NSCLC. (Figure 3B and Supplementary Figure 2). Meanwhile, we found the correlation between the 54 genes overlapping with *Epimedium* and LUAD and LUSC was higher than the correlation between the random genes in LUAD and LUSC (Wasserstein Distance =0.0559) (Figure 3C). Taken together, these data suggested that the potential targets of polypharmacology molecules of *Epimedium* have the potential antitumor effect on the TME for the treatment of NSCLC.

### Mechanism of polypharmacology molecules of *Epimedium* targeting TME for the treatment of NSCLC

**Figure 4:**
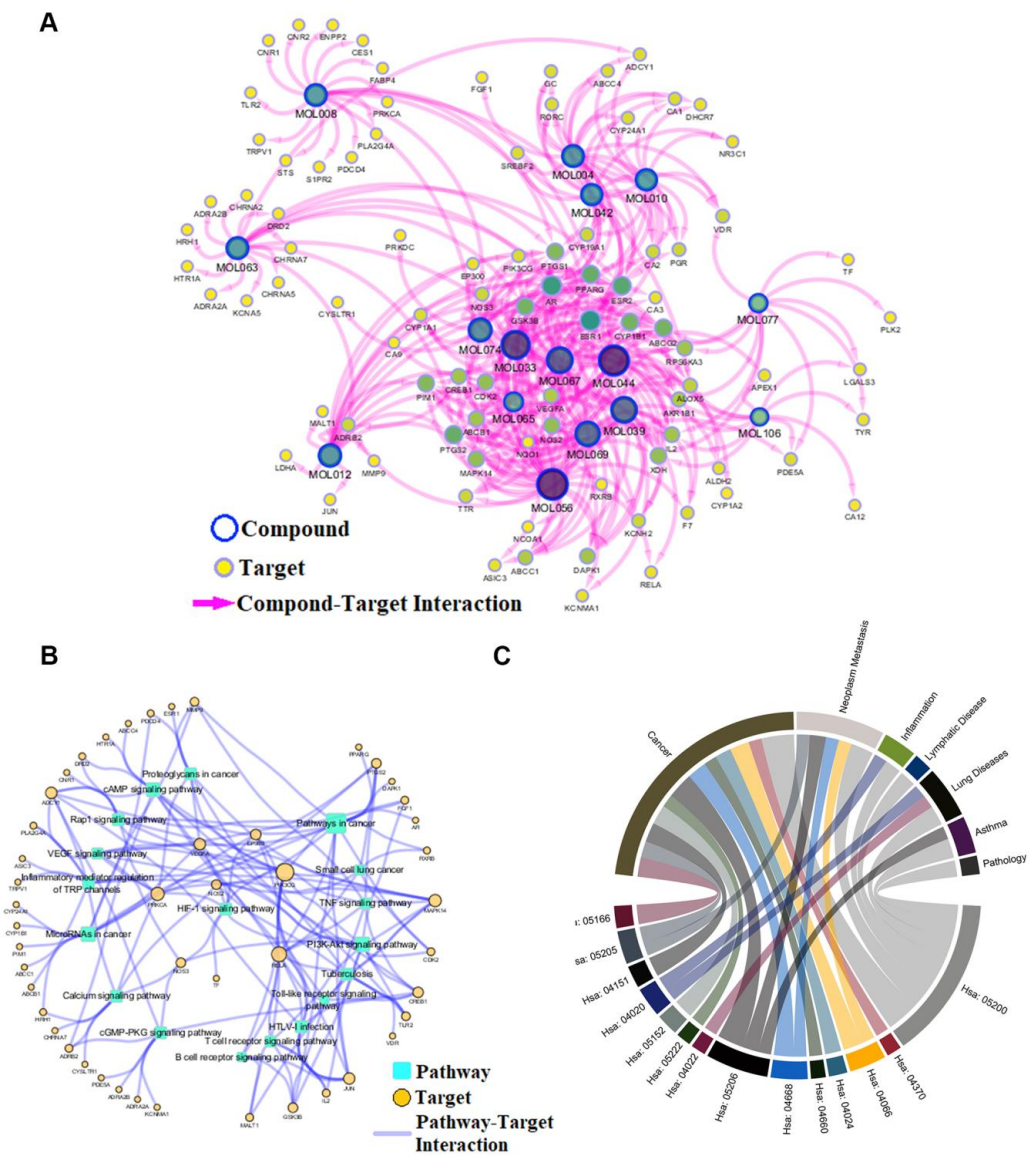
**Network analysis.** (**A**) C-T network. (**B**) T-P network. Yellow nodes represent potential targets; blue circles represent active ingredients of *Epimedium*; squares represent targets-related pathways; and edge represents the interaction between them. Node size is proportional to its degree. (**C**) P-D chord diagram. The circle represents the selected pathways and diseases, and the color of the bands within the circle reflects the relationship between the pathways and the diseases.

**Figure 5:**
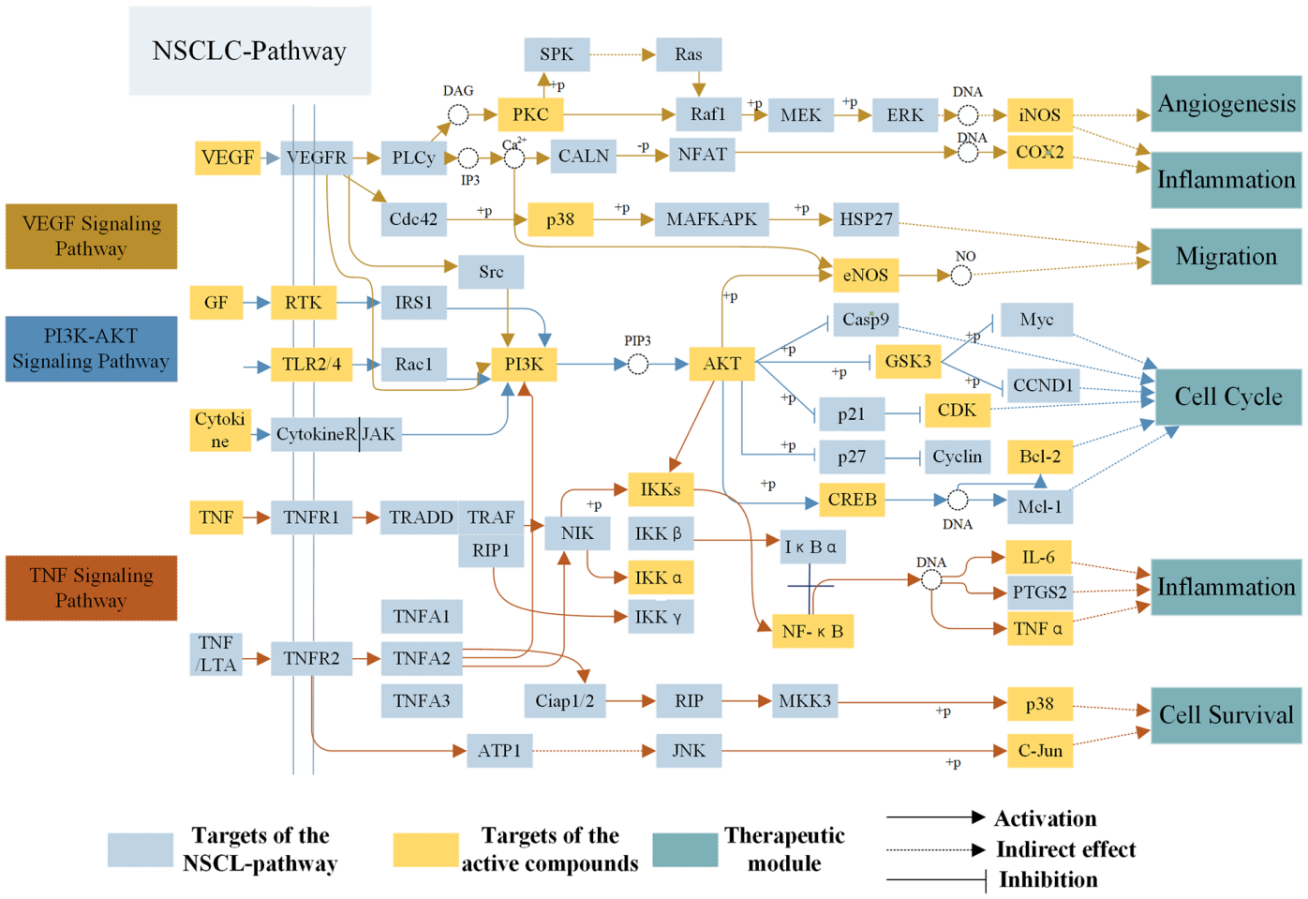
**The NSCLC-pathway.** Distribution of protein targets of *Epimedium* on the compressed ‘NSCLC-pathway’. Three pathways (VEGF, TNF, PI3K-AKT Signaling Pathway) form the compressed NSCLC pathway. Yellow rectangle remark targets on the NSCLC pathway, blue rectangle represent targets of active compounds and dark blue rectangle represent therapeutic modules. Solid arrows indicate activation, T-arrows indicate inhibition and dotted arrows indicate indirect effect.

To reveal action mechanism of the multi-compound and multi-target features of *Epimedium* targeting the TME in response to NSCLC, we analyzed the interaction of potential compounds, targets, pathways and diseases by compound-target (C-T) network, target-pathway (T-P) network and pathway-disease (P-D) chord diagram (Figure 4A–4C). For example, in the C-T network, ICT, kaempferol and sitosterol target proteins associated with inflammation, cancer cell apoptosis and migration in the TME [37, 41–43], such as COX2, IL-6, GSK3β, CDK2 and NOS3. For the T-P network, we found that about 22% of targets were involved in TNF, PI3K-Akt and VEGF signaling pathway, while P-D chord diagram showed that TNF, PI3K-Akt and VEGF signaling pathways were significantly enriched in Cancer and Neoplasm Metastasis diseases. Moreover, a "NSCLC-pathway" containing TNF, PI3K-Akt and VEGF signaling pathways was assembled and separated into two represent therapeutic modules (immunization module and tumor related module). (Figure 5).

### Immunization module

At present, inflammation has a close relationship with the onset of tumor, which not only promoted the occurrence and development of tumor, but also participated in various processes of tumor cells proliferation and migration in the TME. As shown in Figure 5, TNF-α, a member of the pro-inflammatory cytokine family, stimulated the recruitment of neutrophils and monocytes to the site of infection and promoted a sustained response to inflammation in the TME [44]. IL-6 is a cytokine that produces a broad range of cellular (macrophages and fibroblasts) and physiological responses upon activation, and that has been implicated in the tumorigenesis and tumor migration of epithelial cancer [45]. ICT (MOL056) could improve the TME by regulating TNF signaling pathway to inhibit inflammatory cells (neutrophils, monocytes, macrophages and fibroblasts) from expressing TNF-α and IL-6 and other inflammatory factors, thereby preventing the development of tumors. The results showed that the polypharmacology molecules of *Epimedium* played an anti-inflammatory role in the treatment of NSCLC by targeting the TNF signaling pathway.

### Tumor related module

As shown in Figure 5, targets targeting TME are mapped to PI3K-AKT and VEGF signaling pathway, which control tumor development by inhibiting cell proliferation and cell migration. For example, in PI3K-AKT signaling pathway, Bcl-2 and Bax are members of Bcl-2 family group, and Bcl-2 has antiapoptotic effect while Bax functions as pro-apoptotic marker [46]. Studies have shown that ICT (MOL056) could act on the PI3K-AKT signaling pathway to down-regulate the expression of Bcl-2 and up-regulate Bax, which promotes the apoptosis of lung cancer cells in the TME, thereby achieving the purpose of treating lung cancer [47]. CDK2 is a main cell cycle protein which could promote the transition from G1 to S phase, thereby promoting the proliferation of tumor cells and the development of tumors [48]. These results confirm that CDK2 is a potential therapeutic target of PI3K-AKT signaling pathway to promote tumor cells apoptosis in the treatment of NSCLC. Furthermore, tumor vascularization is an important process of tumor growth, invasion, and metastasis in the TME. It has been reported in the literature that the sitosterol (MOL042) was capable of regulating the expression of some proteins in the VEGF signaling pathway to prevent tumor cells migrate [49], such as eNOS, PKC and P38, etc. eNOS, which is produced by endothelial cells, is a key factor in regulating vascular function because it produces nitric oxide that causes vasoconstriction. And controlling its expression inhibits tumor cells migration [50]. Accordingly, the above analysis indicated that the polypharmacology molecules of *Epimedium* could achieve pro-apoptosis and inhibit tumor cells migration by targeting the PI3K-AKT signaling pathway and VEGF signaling pathway. To sum up, we surmised that the action mechanism of polypharmacology molecules of *Epimedium* in treating NSCLC might be via targeting TNF, PI3K-Akt and VEGF signaling pathways in the TME to regulate multiple targets in multiple cells and exert effects of anti-inflammatory, promote apoptosis and anti-migration.

### *In vivo*, the anti-NSCLC effect of ICT

**Figure 6:**
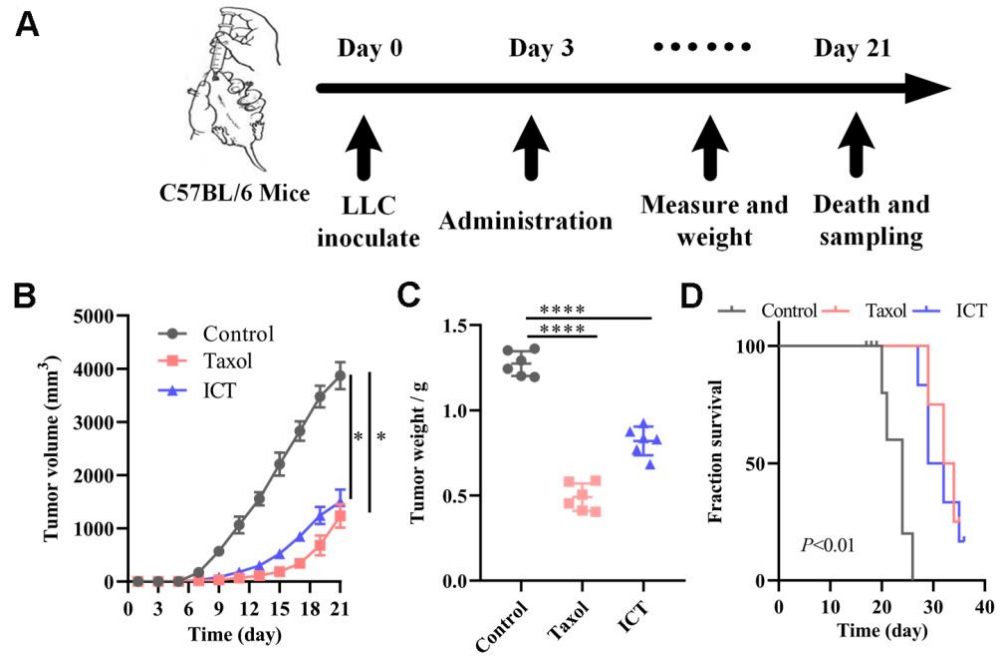
**ICT treatment inhibited tumor growth in mice.** (**A**) *In vivo* experimental treatment plan flow diagram. (**B**) The tumor volume in control, positive control, and ICT- treated mice was measured once every two days using digital calipers. (**C**) Weight of LLC tumors following sacrifice in mice subjected to daily administered for 21 days, and their controls. (**D**) Kaplan-Meier survival curves of mice which were inoculated with LLC cells. All data represent the means ± SEM of six mice. *P < 0.05, ****P < 0.0001 vs control group.

Based on the above systems pharmacology analysis, we selected ICT, a major active ingredient of *Epimedium*, possessing the highest degree in the C-T network and good pharmacological properties to verify the therapeutic effect of polypharmacology molecules of *Epimedium* on NSCLC. The LLC tumour models were constructed as stated in “Materials and methods” (Figure 6A). 5×10^5^ LLC cells were injected subcutaneously in the left axilla of C57BL/6 mice. After three days, mice received daily intragastric administration of ICT or daily intraperitoneal administration of Taxol or daily intraperitoneal administration of diluent for 18 sequential days. Tumor volume and mice body weight were measured once every two days when diameter of tumor reached 5×5 mm after 6 days. At day 21, mice were sacrificed by cervical dislocation for molecule analysis. As shown in Figure 6B, 6C, we statistically analyzed the tumor volume and weight of mice and found that the ICT group and positive control group significantly inhibited the growth of tumor compared with the control group. Besides, as shown in Figure 6D, the ICT group and positive control group exhibited a significant increase in survival, compared with the control group. And no significant loss of mice weight was observed during the experiment (Supplementary Figure 3). These data suggested that ICT could inhibit tumor growth of LLC in mice and extend the life cycle of mice.

### ICT altered the TME by increasing CD8+ T cell infiltration

**Figure 7:**
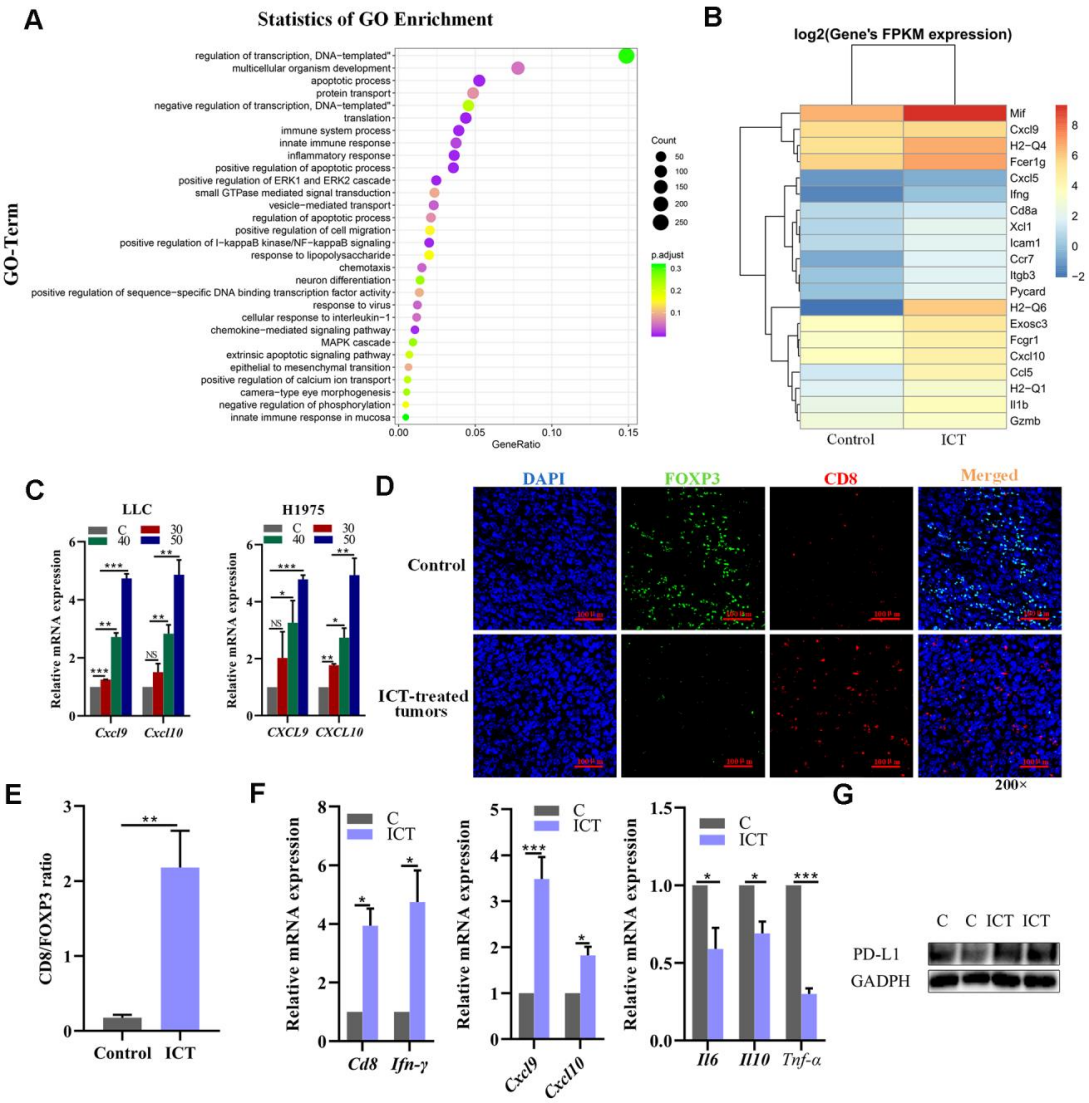
**Analysis of increased CD8+ T cell infiltration in ICT-treated tumors.** (**A**) GO enrichment analysis of differential genes between ICT-treated tumors and control group by ggplot2. The color represents the different adjusted *P*-values < 0.05, while the size of the circle represents the count. GeneRatio indicates the number of differential genes located in the GO/the total number of genes located in the GO, and the larger the GeneRatio, the higher the degree of GO enrichment. (**B**) The heatmap of the expression of different genes in different samples, where the abscissa is the sample and the ordinate are the gene. The different colors indicate different gene expression levels, and the color from blue to white to red indicates the expression level from low to high. (**C**) CXCL9 and CXCL10 mRNA expression in the mouse LLC and the human H1975 lung cancer lines were determined 24 h after treatment with ICT and the expression level increased at a dose-dependent manner. (**D**) Representative immunofluorescence staining images of CD8+ T cells and FoxP3+ regulatory T-cells infiltration in ICT-treated tumor and control group tissue sections. CD8+ T cells were stained red (CD8), FoxP3+ regulatory T-cells were stained green (FOXP3) and nuclei stained blue (DAPI). (**E**) Quantitative analysis of the ratio of CD8/FOXP3 in the figure (**D**). (**F**) T cell marker factors (CD8 and IFN-γ), chemokines (CXCL9 and CXCL10), and inflammatory factors (IL6, IL10 and TNF-α) mRNA expression were determined in ICT-treated tumor tissues by RT-PCR. *P < 0.05, **P < 0.005, ***P < 0.001, ****P < 0.0001 vs control group. (**G**) Protein expression of PD-L1 was determined in control (**C**) and ICT-treated tumor tissues by immunoblotting.

To further explore the mechanisms of ICT in the treatment of NSCLC, we performed transcriptome sequencing for ICT-treated tumor (one sample) and control-treated tumor (one sample) (Supplementary Table 4). We used R LPEseq package to detect the differentially expressed genes (DEGs) with the thresholds of the p-adjust value ≤ 0.05 and |log2 fold change |≥1. LPEseq resulted 1779 upregulated and 596 downregulated DEGs in ICT-treated tumor, respectively. Surprisingly, we founded the 1779 upregulated DEGs in ICT-treated tumors were mainly enriched in immune system process, inflammatory response, NF−kappaB signaling and innate immune response, which indicated that ICT could target TME regulation immune pathway in murine tumor models (Figure 7A). A more detailed analysis revealed upregulation of genes involved in immune activation and immune cell homing, such as antigen presentation (MHC I and II), chemotaxis (CXCL9 and CXCL10) and immune cell homing (ICAM1) (Figure 7B). Then, we further confirmed that ICT treatment increased CXCL9 and CXCL10 secretion in the mouse LLC and the human H1975 lung cancer cell lines by RT-PCR (Figure 7C). Studies have shown that increased expression of T cell chemokines (such as CXCL9 and CXCL10) in the TME was closely related to immunotherapy [51].

In addition, T cell chemokines promote increase of T cell infiltration and improved patient survival [52–54]. Therefore, we asked whether there were greater CD8+ T cells infiltration in ICT-treated tumors using immunofluorescence. As shown in Figure 7D, 7E, we observed a significant increase in the ratio of ICT-treated tumor-infiltrating CD8+ T cells to FoxP3+ regulatory T-cells compared to the control group, indicating that ICT activated the antitumor response of cytotoxic T lymphocytes. To substantiate this result, we further examined the expression levels of T cell marker factors and chemokines in tumor tissues using RT-PCR. We found that whole-tumor gene expression of T cell marker factors (CD8 and IFN-γ) and chemokines (CXCL9 and CXCL10) were significantly enhanced after ICT treatment, while inflammatory factors (IL6, IL10 and TNF-α) were reduced (Figure 7F). In the previously studies, the ICT has been used in the advanced hepatic cell carcinoma treatment via activating CD8^+^ T cells function in TME by downregulating PD-L1 expression [55]. To examine whether the PD-L1 pathway is blocked by ICT in LLC tumour tissues, we examined the PD-L1 expression by immunoblotting, but found that ICT upregulated the PD-L1 expression (Figure 7G), which may be induced by upregulated IFNG in TME [56]. The different effects of ICT on PD-L1 may be caused by tissular differences. These suggest that ICT triggers the antitumor immunity in lung cancer by regulating inflammatory signaling pathways and increases immune chemokines expression instead of downregulating PD-L1 pathway as in hepatic cell carcinoma.

### Pro-apoptosis and anti-inflammatory effects of ICT on lung cancer cells

**Figure 8:**
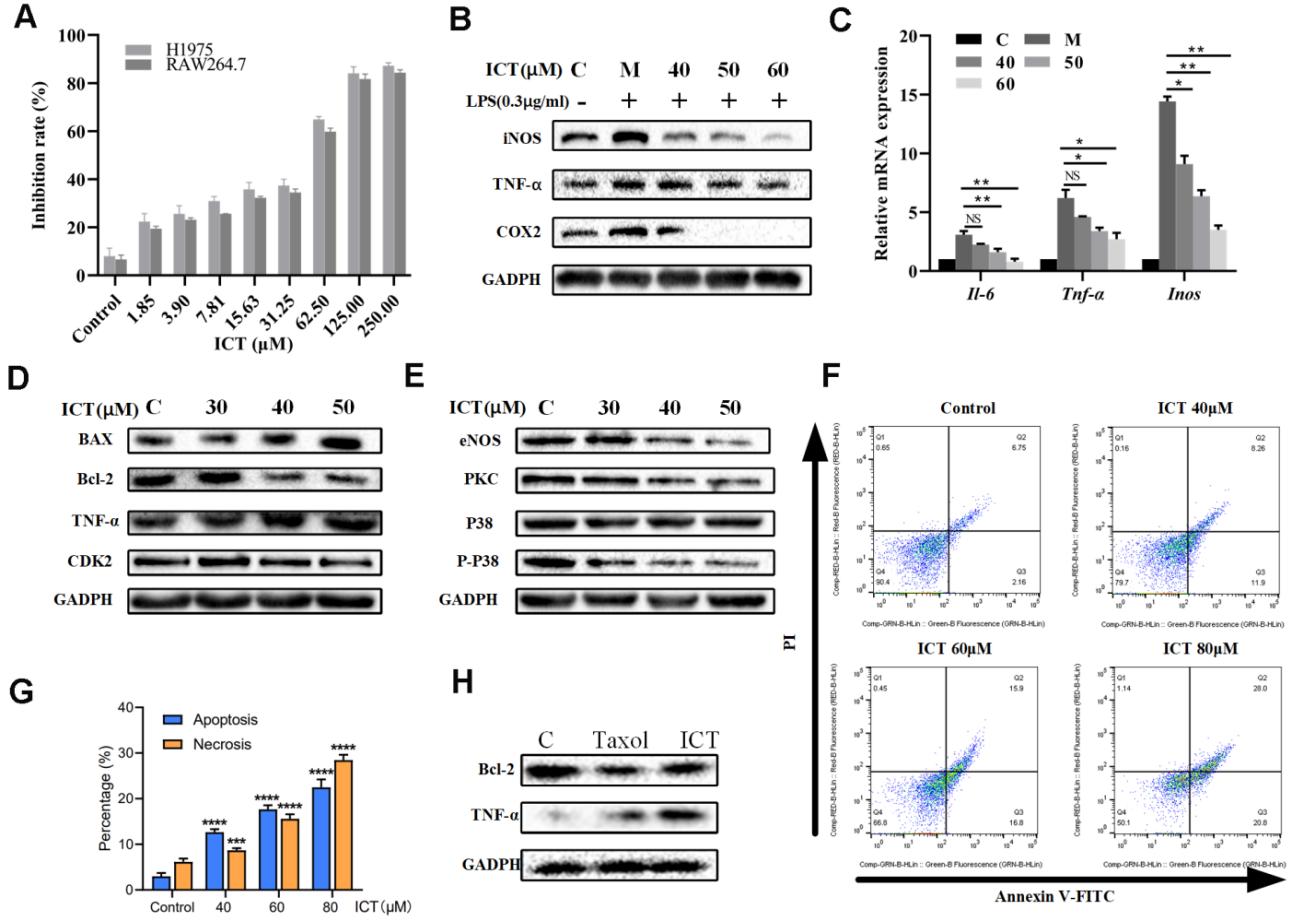
**The effect of ICT on lung cancer cell lines.** (**A**) Inhibition rates of ICT on H1975 and RAW264.7 cells. After incubated 24 h, H1975 and RAW264.7 cells viability was measured by CCK-8 assay after treated with control or ICT for 48h. (**B**) The expression of inflammatory factors (iNOS, TNF-α and COX-2) was measured by western blotting. (**C**) mRNA expression of inflammatory cytokines (IL-6, TNF-α and iNOS) was determined by RT-PCR. ICT reduced mRNA expression of IL-6, TNF-α and iNOS in LPS-induced RAW264.7 cells. *P < 0.05 vs LPS group, **P < 0.005 vs LPS group. (**D**) Western blotting showed the expression of classical apoptotic proteins (TNF-α, Bcl-2, Bax, CDK2) in H1975 cells. (**E**) Western blotting showed that ICT down-regulated the expression of migration proteins (eNOS, PKC, P38) in H1975 cells. (**F**) Apoptosis in H1975 cells was assessed after 24 h of treatment with ICT (0, 40, 60 and 80μM) by Annexin V-FITC/PI binding and measured by flow cytometry analysis. Numbers indicate the percentage of cells in each quadrant. The number of lower right quadrant and upper right quadrant represent the percentage of apoptosis and necrosis, respectively. (**G**) Bar plot represents the percentage of apoptosis and necrosis induced by ICT in flow cytometric image (**F**). *P < 0.05, **P < 0.005, ***P < 0.001, ****P < 0.0001 vs control group. (**H**) Western blotting showed that the expression of extrinsic protein TNF-α increased and the expression of internal protein Bcl-2 decreased in ICT-treated tumor tissues. *P < 0.05, **P < 0.005, ***P < 0.001, ****P < 0.0001 vs control group. GAPDH was used as the loading control.

In view of effective effects of ICT on LLC tumor-bearing mice, we have further verified *in vitro*. Previously, it was reported ICT potently inhibited growth of prostate cancer PC-3 cells, breast cancer cells and hepatoma HepG2 cells [57, 58]. We decided to examine the effect of various concentrations of ICT on the survival rate of H1975 cells and RAW264.7 cells by CCK-8 analysis. As shown in Figure 8A, the IC_50_ values of ICT against H1975 cells and RAW264.7 cells were 53.92 μM and 55.45 μM, respectively. Then, according to the IC_50_ values, we selected the concentration of ICT at 50, 40, 30 μM and 60, 50, 40 μM for subsequent experiments on H1975 cells and RAW 264.7 cells, respectively.

The NSCLC-pathway showed that Epimedium and ICT can target tumour-promoting inflammation signaling pathways. As a major component of the leukocyte infiltrate in tumors, macrophages are crucial drivers of tumour-promoting inflammation which impairs CD8+ T cells function [59]. Also, we found that over half of ICT targets (18/32) significantly correlate with abundance of macrophages in TCGA LUAD patients (Figure 2C). Thus, we speculated ICT might promote CD8+ T cell mediated antitumor immunity by suppressing inflammation derived from macrophages. To test this, RAW264.7, a typical macrophage-lineage cell line, was used to validate the anti-inflammatory effects of ICT on macrophages. The results presented in Figure 8B showed that level of TNF-α, iNOS and COX-2 in LPS stimulated culture were significantly higher than that in normal control, which implied the successful establishment of a model of inflammation. When RAW264.7 cells were treated with different concentrations of ICT, the LPS stimulation induced high expression of TNF-α, iNOS and COX-2 were remarkably inhibited. To further characterize the anti-inflammatory effect of ICT in modulating the TNF signaling pathway, we analyzed the expression of TNF-α, IL-6 and iNOS by RT-PCR, and found that ICT suppressed the LPS-induced mRNA expression of TNF-α, IL-6 and iNOS (Figure 8C). These results suggested that ICT mainly regulated the expression of inflammatory factors *via* the TNF signaling pathway to change the TME.

Moreover, to determine the proliferation-inhibition effect of ICT on H1975 cells, we measured the changes of extrinsic cell apoptosis protein TNF-α, anti-apoptotic protein Bcl-2, pro-apoptotic protein Bax and cell cycle regulatory proteins CDK2 in PI3K-AKT signaling pathway and angiogenic proteins eNOS, PKC and P38 in VEGF signaling pathway under the action of ICT. As shown in Figure 8D, in H1975, the expression of extrinsic cell apoptosis signal TNF-α is increased in a dose-dependent manner after ICT treatment. Note that, for internal signal, we also observed that ICT reduced the levels of Bcl-2 and CDK2 while increased Bax expression (Figure 8D). And eNOS, PKC and P38 also presented a decreasing trend after ICT treatment (Figure 8E). Thus, these results show that ICT triggers apoptosis through both of extrinsic and intrinsic apoptosis pathways *in vitro*. Furthermore, by flow cytometry analysis, compared with control group, ICT dramatically triggered apoptosis in human lung cancer H1975 cells (Figure 8F, 8G). According to the results of flowcytometry, we counted the percentage of apoptosis (lower right quadrant, Q3) and necrosis (upper right quadrant, Q2) induced by ICT. Compare with the control group, at relative low concentration (40 μM) ICT, ICT induced drastically increase of apoptosis (fold change = 4.2(12.65%/2.99%)), but not necrosis (fold change = 1.4 (8.69%/6.13%)) (Figure 8G). However, at high concentration (80 μM), ICT induced copious necrosis (28.46%) which overtaken apoptosis (22.47%). These results suggested that ICT induced apoptosis at the relative low concentration but necrosis at high concentration. Finally, we have detected the apoptosis related proteins in the ICT treated tumour tissue. Compared with the control, we found that the expression of extrinsic protein TNF-α increased and the expression of internal protein Bcl-2 decreased in ICT-treated tumor tissues (Figure 8H). To sum up, the experiments *in vitro* confirmed that ICT could exert the effect of anti-inflammation and pro-apoptosis by regulating the expression of critical proteins in our integrated NSCLC-pathway, achieving the anti-NSCLC effect.

## DISCUSSION

With the development of the field of tumor immunotherapy, immunotherapy that enhances the ability of cytotoxic T lymphocytes by targeting TME to kill tumor cells has been confirmed in a variety of human malignancies [60–62]. However, NSCLC patients are resistant to many targeted therapeutic drugs and have poor prognosis. Given the development of multi-target natural products that can target TME to regulate tumor biological networks and pathways, enhancing antitumoral responses of CD8+ T cells and making the efficacy more durable. Therefore, it is necessary to conduct additional studies for natural products to improve antitumor immune effect and optimize patient selection. In this paper, we used systems pharmacology approach to reveal the action mechanism of polypharmacology molecules of *Epimedium* targeting TME for NSCLC.

With the aid of favorable pharmacokinetic characteristics, 16 active ingredients of *Epimedium* were obtained. Among them, it was reported that some vital compounds of *Epimedium* such as ICT, kaempferol and sitosterol have antitumor activity [63–65]. And 89 potential targets were predicted by bioinformatic algorithms. Association analysis of diseases and target function enrichment analysis together displayed that the targets were closely related to neoplasms and processes of inflammation and immune.

In conclusion, the systems pharmacology models integrated the multiple methods including the active compounds screening, target predicting, network pharmacology analysis and onco-immune interacting to predict the natural products that target TME. Applying the systems pharmacology, we successfully revealed the ICT enhanced the infiltration of CD8+ T cells in TME via multiple pathways, which shown the accuracy and reliability of systems pharmacology. According to these results, we speculate ICT could be used to treat the NSCLC patients with low infiltration of CD8+ T cells, which provided the basis for the sponsors to set inclusion/exclusion criteria in future trial design. In addition, we consider that Epimedium and ICT might sensitize the cancers to anti-PD-1/PD-L1 or anti-CTLA4 via increasing the infiltration of CD8+ T cells, since immune checkpoint blockade therapies often fail in the patients with poor T cell infiltration. In the future, we believe that systems pharmacology is a potential framework for rationally designing combination therapies with immune checkpoint blockade through screening the lead compounds that target pathways determining the resistance of the immune checkpoint blockade.

## MATERIALS AND METHODS

### Pharmacokinetic screening

All chemical components of *Epimedium* were retrieved from the literature and uploaded to the TCMSP (http://lsp.nwu.edu.cn/tcmsp.php) [66]. As a result, 130 ingredients were got for the *Epimedium*, which molecular structures were saved as mol2 format for subsequent analysis.

Based on the compounds obtained above, we used the *in silico* to screen potential active molecules with favorable pharmacokinetics properties that were potent anti-NSCLC. ADME-based screening criteria, i.e. OB ≥ 30%; DL ≥ 0.18; HL ≥ 4 h. The filtered active compounds must simultaneously satisfy three conditions.

### Target fishing

In this work, we used SEA (http://sea.bkslab.org/) [67], WES algorithm [68] and SysDT [69] to understand the nature of the compound-target interaction. Among them, the WES computational model was introduced to detect drug direct targets of the active ingredients based on a large-scale of drug target relationships [68]. SysDT model, based on random forest (RF) and support vector machine (SVM), was utilized to explore potential targets of drugs, which could comprehensively determine the distribution of compound-target interaction. The compound-target interactions with SVM score ≥ 0.8 and the RF score ≥ 0.7 were selected for further research [69]. Then, we mapped targets to the Uniprot (http://www.uniprot.org), unifying their names and organisms. Normalized compound targets were mapped to the CTD database (http://ctdbase.org/) [70], Therapeutic Target Database (TTD, http://database.idrb.cqu.edu.cn/TTD/) [71], and Pharmacogenomics Knowledgebase (PharmGKB, https://www.pharmgkb.org/) [72] to obtain their corresponding diseases and screen out potential targets related to NSCLC.

### Target-associated diseases analysis

To illustrate the relationship between predicted potential targets and treatment of NSCLC, we mapped these targets to the CTD to obtain their related diseases, and classified these diseases into 12 categories based on their direct or indirect effects. Then, we constructed a radar chart to analyze the degree of association between the target and the disease.

### GOBP analysis

In order to further probe the meaningful functional annotation of the potential targets, we performed the GO enrichment analysis by linking the targets to ClueGO (Cytoscape plugin). Only GO terms with *P*-value ≤ 0.05 are selected. The Enrichment Map [73], a *Cytoscape* plug-in, was implemented as a freely available and open source for visualization and analysis software. We applied the GSEA (Gene-Set Enrichment Analysis) to analyze enrichment significance of gene-sets, and then used to find enriched GO gene-sets. Only gene-sets passing conservative significance thresholds (*P*-value < 0.005, False Discovery Rate (FDR) < 1%) were screened out for display in the Enrichment Map, illuminating the biology processes of the lists of genes.

### Correlation between targets and clinical features in LUAD patients from TCGA

We correlated the targets to the LUAD clinical features including ten immune phenotypes, disease stage and overall survival time. The RNA-seq data and information of clinical stage and overall survival time of LUAD in TCGA were download by R TCGABiolinks package. The ten antitumor immunity-related phenotypes involve the proportion of major classes of immune cells (leukocyte, T cells CD8, activated NK cells, activated dendritic cells, Tregs, macrophages M2), TCR richness and the TIL regional fraction on tissue image, which are implicated in regulating tumor responses to host immunity. These data were obtained from the curated methods that were described in llya Shmulevich et al. [38]. We correlated the mRNA level (log2(TMP+1)) of targets with these phenotypes using Pearson's correlation coefficient (PCC). The p-values of PCC were adjusted by Benjamini–Hochberg methods. To correlate targets expression with overall survival, we evaluated the target set expression level for each of LUAD patient by PLAGE (Pathway Level Analysis of Gene Expression) approach, which translates gene expression levels into 'pathway activity' levels using singular value decomposition [74]. Kaplan-Meier plots summarized the results from correlation analysis between PLAGE expression level and patient survival. Patients were divided based on level of PLAGE activity into one of the two groups "low activity" (under cut off) or "high activity" (over cut off). The cut off was set to minimize the p-value of log rank test and guarantee the fraction of "low activity" or "high activity" group that should never go below 20%. To correlate targets with clinical staging, we normalized targets expression by z-score transformation and averaged the normalized expression by tumour stages (stages I–IV).

### Differentially expression gene analysis

Publicly available gene expression profile of TCGA-LUAD and TCGA-LUSC were downloaded from the Genomic Data Commons (GDC) Data Portal and pre-processed via the *TCGAbiolinks* R package [40]. Then, the DEGs of LUAD and LUSC were filtered using the *Limma* pipeline in the *TCGAbiolinks* with the |log2FC| cutoff of 1 and FDR cutoff of 0.01. Finally, we plotted a Venn diagram and analyzed overlap ratio between the potential targets of the *Epimedium* active components and the DEGs of LUAD and LUSC. Furthermore, we further calculated pairwise correlation of 89 potential genes of *Epimedium* active components by the Pearson method and constructed a gene-gene correlation matrix and heatmap. Subsequently, we randomly acquired 53 genes and calculated the correlation between them. Meanwhile, we mapped the distribution diagram of random intergenic correlations and *Epimedium* intergenic correlation, and calculated Wasserstein distance between the two distribution diagrams.

### Network construction

To directly reflect the relationship between compounds, targets, pathways and diseases, we constructed C-T network and T-P network by *Cytoscape 3.7.0* [75], which is a popular bioinformatics package for biological network visualization and data integration. In the generated network, the nodes represent compounds, targets and pathways, and the interactions between them were represented by edges. The topological properties of these networks were analyzed using the Network Analysis plugin and *CentiScaPe 1.2* of *Cytoscape 3.7.0*.

In addition, we collected genes of the *Epimedium* active components in the pathways and the diseases, and counted the number of common genes between them. The pathways and diseases with more than 3 overlapping genes were connected, and a chord diagram of P-D interactions was plotted by using the *Circos* (*Circlize* package in R v3.4) [76].

### Pathway construction

To further explore how the active compounds exert their pharmacokinetic effects by modulating targets in pathways, an incorporated “NSCLC-pathway” was assembled based on the latest information on NSCLC pathology. In short, the targets were firstly mapped to DAVID and the pathways associated with NSCLC were obtained and screened from the KEGG database (http://www.genome.jp/kegg/) [77], and which were then manually integrated into the NSCLC-pathway based on pathological and clinical data.

### Reagents

ICT was purchased from Shanghai Yuanye Bio-Technology Co., Ltd (Shang Hai, China). A stock solution of ICT (100mM) was dissolved in DMSO for *in vitro* experiments, stored at 4° C and further diluted to appropriate concentrations before use. The final concentration of DMSO was < 0.1% in culture and insured there was no effect on cell viability. Dulbecco’s modified Eagle’s medium (DMEM) and RPMI-1640 (Gibco BRL, USA), Dimethyl sulfoxide (DMSO) (Sigma, USA), CCK-8 (BestBio, Shanghai, China), fetal bovine serum (FBS) (Gibco BRL, USA), Qproteome™ Mammalian Protein Prep Kit (Qiagen, Germany), BCA Protein Assay Kit (Beyotime, Shanghai, China), FITC Annexin V apoptosis kit (BD Pharmingen, Franklin Lakes, NJ, USA), Trizol reagent (Takara BioCo., China), MuLV reverse transcriptase and Oligo-dT primers (Takara BioCo., China), SYBR green PCR Master Mix (Takara BioCo., China) were obtained as indicated. We used the following primary antibodies: GAPDH (1:1000); Bcl-2 (1:1000); Bax; GSK3β(1:8000); CDK2 (1:2000); iNOS (1:1000); eNOS (1:1000); PKC(1:50000); p38 (1:1000); p-p38 (1:1000); COX2 (1:1000); TNF-α (1:1000); and Goat Anti-Mouse IgG H&L (HRP) were obtained from Abcam.

### Animal and drug treatment

Female C57BL/6 mice (5-6 weeks, 18–22g) were obtained from the Comparative Medicine Centre of Yangzhou University (Yangzhou, China). They were maintained under specific pathogen-free conditions at the Institute of Laboratory Animals, Jiangsu Kanion Pharmaceutical, Co., Ltd. and used under protocols approved by the respective Institute of pharmacology and toxicology institutional review board (IRB), IACUC No. (2019040401).

The animals were maintained in a pathogen-free environment at 25 ± 1° C and humidity of 55 ± 5% for 1 week, 12 h light/dark cycle and free access to food and water under a standard specific pathogen free (SPF) condition. LLC cells suspended in phosphate-buffered saline (PBS). Each mouse was subcutaneously inoculated with approximately 5×10^5^ LLC cells to the left axilla. On the third day (after inoculation), mice were randomly divided into six groups: two groups of control (n = 6/group, intraperitoneal injection of diluent); two groups of positive control (n = 6/group, daily intraperitoneal administration of Taxol, 15mg/kg, Yuan ye, Shanghai) and two groups of ICT (n = 6/group, daily intragastric administration of ICT, 15mg/kg, Yuan ye, Shanghai). Among them, three groups (control group, positive control group and ICT group) were used for the analysis of tumor volume and weight. When the tumor reaches 5×5 mm, the size of the tumor is measured by digital caliper and body weight recorded once every two days until the tumor is larger than 20 mm×20 mm, then the mouse is killed and recorded as death. Tumor volume was measured along the longest orthogonal axes and calculated as volume = (length×width^2^)/2, where width was the shortest measurement. On the 21 days, the mice were sacrificed by cervical dislocation, and the tumor was taken out for weight, observation and analysis. While the other three groups (control group and medication group) were used for survival analysis.

### Transcriptome sequencing

Total RNA was extracted using Trizol reagent (Invitrogen, CA, USA) following the manufacturer's procedure. The total RNA quantity and purity were analysis of Bioanalyzer 2100 and RNA 1000 Nano LabChip Kit (Agilent, CA, USA) with RIN number > 7.0. Poly(A) RNA is purified from total RNA (5 μg) using poly-T oligo-attached magnetic beads using two rounds of purification. Then the cleaved RNA fragments were reverse-transcribed to create the final cDNA library in accordance with the protocol for the mRNASeq sample preparation kit (Illumina, San Diego, USA). And then we performed the paired-end sequencing on an Illumina Novaseq™ 6000 at the (LC Sciences, USA) following the vendor’s recommended protocol.

We aligned reads of sample ICT-treated tumors and sample control to the UCSC (http://genome.ucsc.edu/) homo sapiens reference genome using HISAT package, which initially remove a portion of the reads based on quality information accompanying each read and then maps the reads to the reference genome. The mapped reads of each sample were assembled using StringTie. Then, all transcriptomes from Samples were merged to reconstruct a comprehensive transcriptome using perl scripts. After the final transcriptome was generated, StringTie and edgeR was used to estimate the expression levels of all transcripts. StringTie was used to perform expression level for mRNAs by calculating FPKM (Fragments Per Kilobase of exon model per Million mapped reads). The differentially expressed mRNAs and genes were detect threshold of |log2(fold change)| >1 and with statistical significance (q-value < 0.05) by R LPEseq package which is developed to implement the analysis of differential expression with a small number of or non-replicated samples [47]. As a result, we identified 2,375 differentially expressed genes (DEGs), including 1779 upregulated and 596 downregulated DEGs in ICT-treated tumor. And the sequencing data were uploaded to our website of Traditional Chinese Medicine Systems Pharmacology Database (TCMSP, https://tcmspw.com/). Finally, the GO enrichment analysis chart of differentially expressed genes was created by clusterProfiler.

### Immunofluorescence

Analysis of CD8^+^ T cells infiltration in ICT-treated tumor tissues by immunofluorescence. Firstly, the tumor tissues were formaldehyde-fixed and paraffin-embedded following standard procedures. Then, paraffin tissue sections of 4 μm thickness were prepared using a paraffin slicing machine (LEICA RM2126) and the sections were roasted overnight at 37° C, dewaxed and rehydrated. They were then sealed with goat serum for 30 min, washed 3-4 times in PBS, and incubated overnight at 4° C with CD8 Polyclonal Antibody (PA5-88265, Thermo Scientific) and FOXP3 Monoclonal Antibody (11-5773-82, Thermo Scientific). After washing 3-4 times with PBS, the Goat anti-Rabbit IgG (H+L) Highly Cross-Adsorbed Secondary Antibody (A-21245, Thermo Scientific) was added dropwise, incubated at 37° C for 1 h, stained with DAPI for 5-10 minutes, washed 3-4 times with PBS, and air-dried. Observed under a confocal microscopy (Nikon A1R).

### Cells and cell culture

Human NSCLC H1975, RAW264.7 and Lewis lung carcinoma (LLC) cells were obtained from Chinese Academy of Sciences Shanghai cell bank. H1975 cells were cultured in RPMI-1640 media complemented with 10% fetal bovine serum (FBS). RAW264.7 and LLC cells were maintained in DMEM with 10% FBS. The all experiments cells were maintained in a humidified atmosphere of 5% CO_2_ at 37° C. The culture medium was substituted fresh medium every 2-3 days and amplified to new culture when the cells reached approximately 80-90% confluency.

### Cell viability assays

The effects of ICT on H1975 cell viability were determined by measuring the metabolic activity (CCK-8 assay). H1975 cells in the logarithmic phase were seeded in 96-well-plates at a density of 8000 per well and cultured for 24 h. After incubated 24 h, cells were treated with various concentrations of ICT (250, 125, 62.5, 31.25, 15.63, 7.81, 3.9, 1.85, 0 μM) for 48 h. RAW264.7 and H1975 cells have the same experimental protocol. Then, 10 μl of CCK-8 assay was added to each well for 4 h incubation at 37° C and 5% CO_2_. And the absorbance was detected by a microplate reader (Molecular Devices, California, USA) at 450 nm. Cells treated with medium containing 1% DMSO was regarded as negative control. Three reduplicate wells were used for each treatment and the experiments were performed three times.

### Inflammation model

RAW264.7 cells (2×10^6^) were cultured in 150 mm culture dish 24 h and treated with the various concentrations of ICT for 2 h. It was then incubated with 0.3 μg/mL LPS for 18 h. The cells were collected at the end of the culture for western blotting assays, which were used as a detection of inflammatory mediators.

### Western blotting

The expression of protein was analyzed using western blotting. Firstly, H1975 or RAW264.7 cells (2×10^6^) were seeded in 150 mm culture dish overnight and treated with the various concentrations of ICT for 24 h. The cells were harvested and scraped, collected by centrifugation and lysed in Qproteome™ Mammalian Protein Prep Kit. The protein concentration was determined using BCA Protein Assay Kit. Secondly, equalized amounts of proteins from each sample separated by SDS-PAGE and transferred onto a PVDF (Millipore, Bedford, MA, USA) membrane. The membrane was washed three times with TBST buffer and blocked with 5% skim milk for 2 h. After washing three times, the membrane incubated with primary antibodies against Bcl-2, Bax, CDK2, eNOS, PKC, p38, p-p38, iNOS, COX2, TNF-α, and GAPDH at 4° C overnight. Finally, the membrane was washed three times again and incubated with a horseradish peroxidase conjugated secondary goat anti-mouse IgG H&L (HRP) for 1 h at room temperature and then washed twice in TBST, once with TBS, afterwards, detected by using the ChemiDoc™ XRS+ Imaging System (Bio-Rad) and labelling were visualized by ImageLab sofware (Bio-Rad). GAPDH was used as the loading control. All of the western blotting experiments were performed at least three times.

### Flow cytometry assays

Cell apoptosis was determined using a FITC Annexin V apoptosis kit according to the manufacturer’s instructions. Following treatment with various concentrations (80, 60, 40 μM) of ICT for 24 h, the cell suspension was prepared using trypsin and centrifuged at 1,000 rpm for 3 min then rinsed with ice-cold PBS. Cells were then resuspended in binding buffer (10 mmol/L HEPES, pH 7.4, 140 mmol/L NaCl and 2.5 mmol/L CaCl_2_) at a concentration of 1×10^5^ cells/ml. Cells were stained with annexin V-FITC and propidium (PI) for 15 min in the dark before analysis by a flow cytometer (Beckman Coulter Inc, Miami, FL, USA). Annexin V^+^/PI^-^ and V^+^/PI^+^ cells were considered as apoptotic cells.

### Quantitative real-time PCR

Gene expression of TNF-α, iNOS, IL-6, IL-10, IFN-γ, CD8, CXCL9, CXCL10 was detected by RT-PCR. Total RNA of cells and tissues was extracted using Trizol reagent according to the manufacturer's instructions. The primer sequences of the selected genes were designed by the Primer3 software (http://frodo.wi.mit.edu/cgi-bin/primer3/primer3_www.cgi) and listed in below: TNF-α, 5′-GCGACGTGGAACTGGCAGAAG-3′ (forward) and 5′-TCCATGCCGTTGGCCAGGAGG-3′ (reverse); iNOS, 5′-TGGAGTCACAGAAGGAGTGGCTAAC-3′ (forward) and 5′-TCTGAC CACAGTGAGGAATGTCCAC-3′ (reverse); IL-6, 5′-TGGAGTCACAGAAGGAGTGGCTAAG-3′ (forward) and 5′-TCTGACCACAGTGAGGAATGTCCAC-3′ (reverse); IL-10, 5′-ACGGCGCTGTCATCGATT-3′ (forward), 5′-GGCATTCTTCACCTGCTCCA-3′ (reverse); IFN-γ, 5′-ATGAACGCTACACACTGCATC-3′ (forward), 5′-CCATCCTTTTGCCAGTTCCTC-3′ (reverse); CD8, 5′-GTCTATATGGCTTCATCCCACA-3′ (forward), 5′-GTTCAGGGTGAGAACGTACTTA-3′ (reverse); CXCL9, 5′-GGCTCGCAGGGATGATTTCAAGAPDH-3′ (forward), 5′-CCAAGTGCTGCCGTCATTTTC-3′ (reverse); CXCL10, 5′-GGAGTTCGAGGAACCCTAGTG-3′ (forward), 5′-GGGATTTGTAGTGGATCGTGC-3′ (reverse). Reverse transcription was undertaken using MuLV reverse transcriptase and Oligo-dT primers. RT-PCR analysis was performed with the SYBR green PCR Master Mix. The relative expression levels of the genes were quantified as cycle time (Ct) values normalized with GAPDH of the same sample.

### Statistical analysis

All data were expressed as the mean ± SD and represent the results of three separate experiments performed in triplicate unless otherwise stated. Student’s t-test was used to evaluate the difference between two groups, and a one-way ANOVA with a post hoc test was used for comparison among three or more groups. In the figures, the standard symbols were used: *P < 0.05, **P < 0.005, ***P < 0.001, ****P < 0.0001 vs control group. *P* < 0.05 was considered to be statistically significant.

## Supplementary Material

Supplementary Figures

Supplementary Tables 1 and 2

Supplementary Table 3

Supplementary Table 4
