# Supplementary material for: Systems pharmacology dissection of Epimedium targeting tumor microenvironment to enhance cytotoxic T lymphocyte responses in lung cancer
Source: Aging (Albany NY). 2021 Jan 17;13(2):2912–40. doi: 10.18632/aging.202410 (PMC7880341; doi:10.18632/aging.202410)
Supplement: Supplementary Figures [file aging-13-202410-s001.pdf]

SUPPLEMENTARY FIGURES

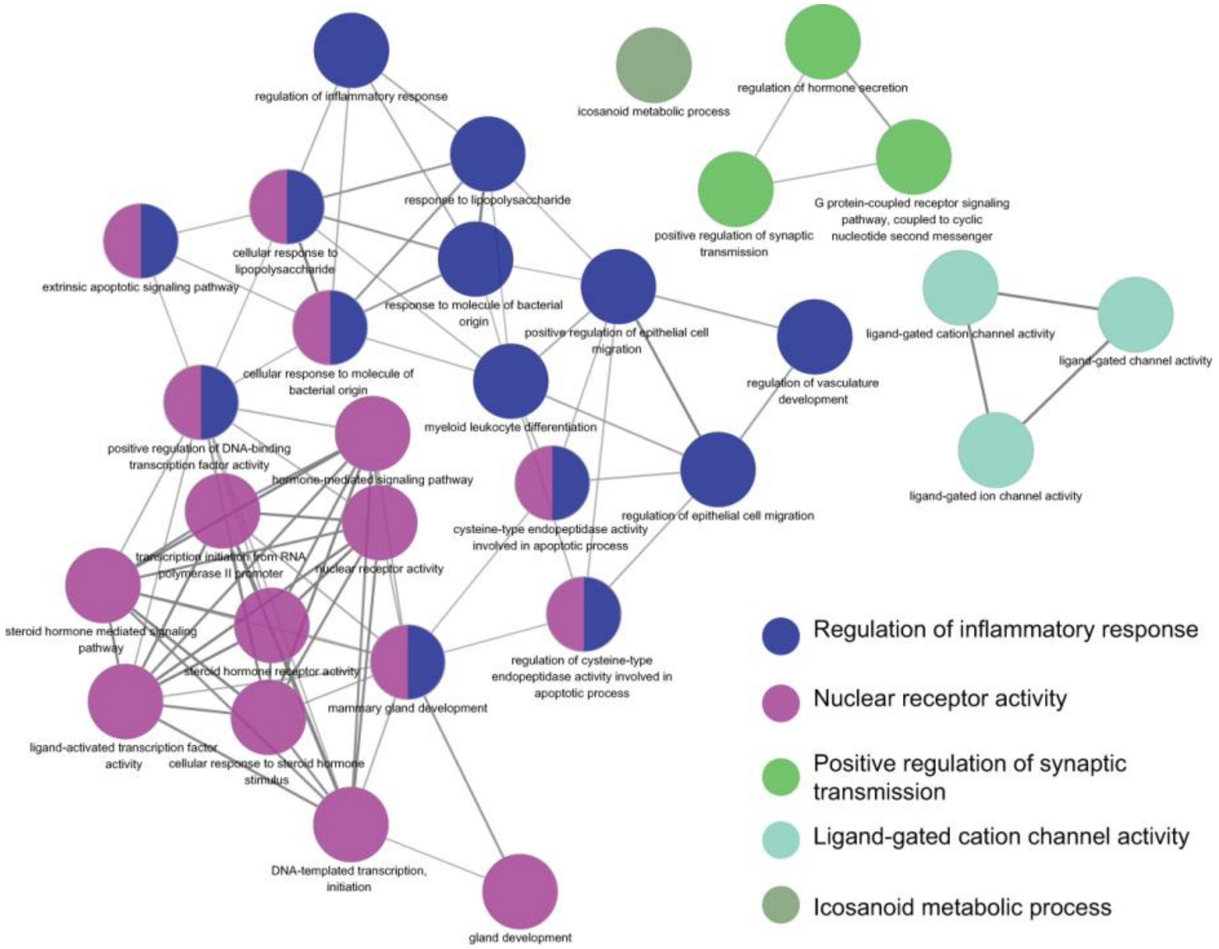

Supplementary Figure 1. The dots represent biological processes associated with potential targets, and the lines represent interrelationships between biological processes.

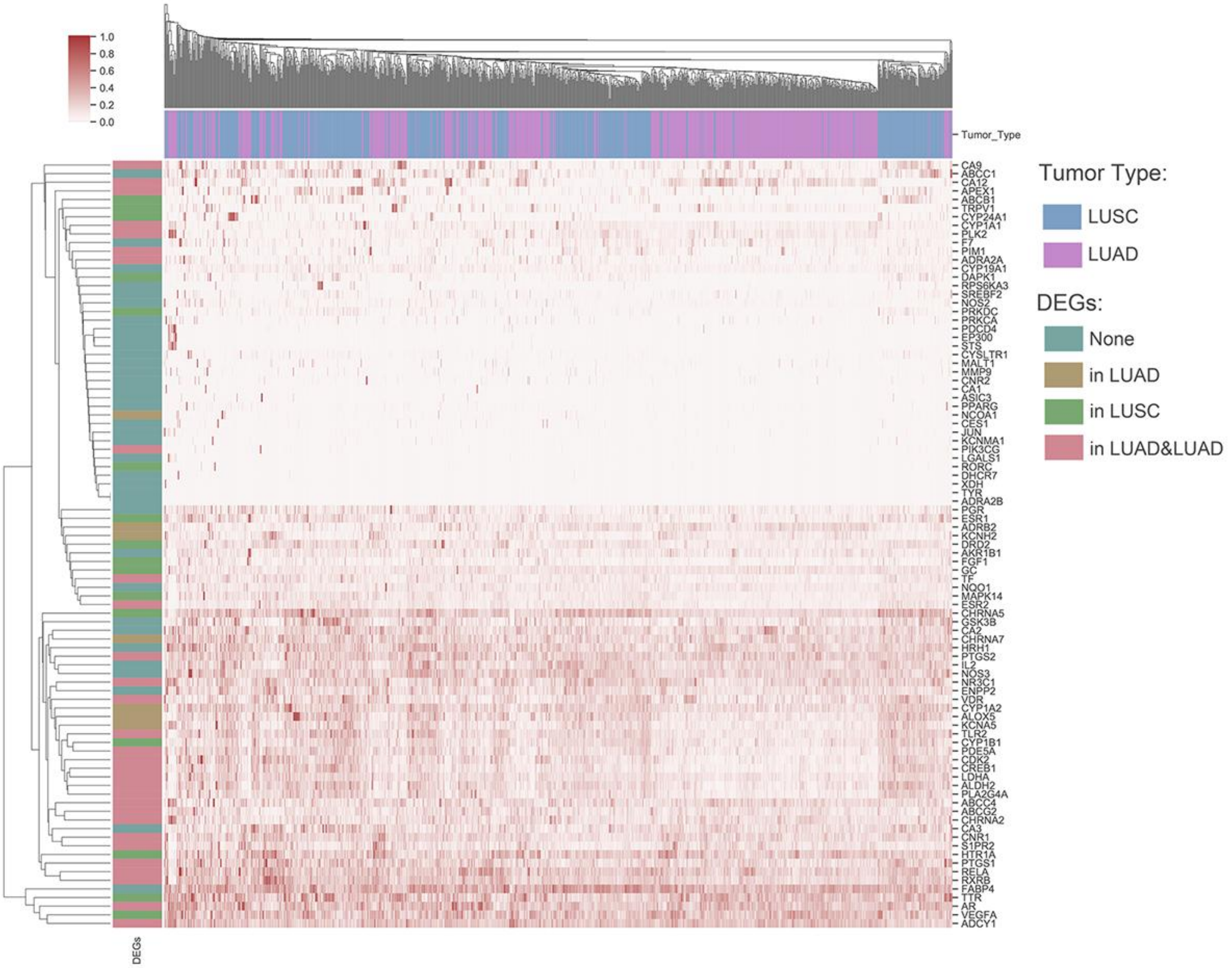

Supplementary Figure 2. Heatmap depicting the expression of potential targets of *Epimedium* active ingredients in LUSC and LUAD with hierarchical clustering.

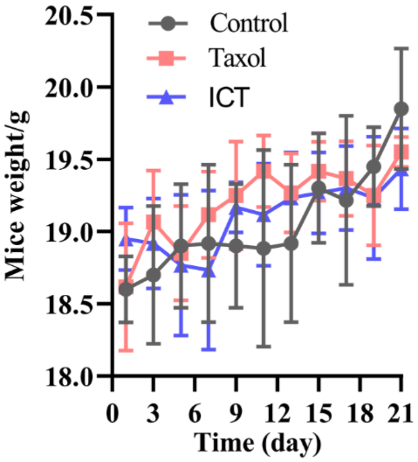

Supplementary Figure 3. At 21 days, there was no significant change in body weight of mice in the control group, positive control group and ICT group.
