# Supplementary material for: Systems pharmacology dissection of Epimedium targeting tumor microenvironment to enhance cytotoxic T lymphocyte responses in lung cancer
Source: Aging (Albany NY). 2021 Jan 17;13(2):2912–40. doi: 10.18632/aging.202410 (PMC7880341; doi:10.18632/aging.202410)
Supplement: Supplementary Tables 1 and 2 [file aging-13-202410-s002.pdf]

SUPPLEMENTARY TABLES

Supplementary Table 1. Candidate targets for each active compound.

MOL-ID	Compounds	Potential targets
MOL056	Icaritin	PDE5A, NOS2, PTGS1, KCNH2, ESR1, AR, PPARG, PTGS2, NOS3, ADRB2, ESR2, MAPK14, GSK3B, CDK2, RXRB, PIM1, F7, NCOA1, KCNMA1, ABCG2, CYP19A1, CYP1B1, CREB1, DAPK1, IL2, RPS6KA3, ALOX5, ABCB1, ABCC1, NQO1, RELA, XDH
MOL044	Kaempferol	ESR1, GSK3B, XDH, ABCG2, DAPK1, KCNH2, PPARG, APEX1, PIM1, NOS2, PTGS1, AR, PTGS2, ESR2, MAPK14, CDK2, PIK3CG, PGR, NOS3, F7, ALDH2, AKR1B1, CA2, CA3, CYP1B1, CYP1A2, CREB1, IL2, RPS6KA3, ALOX5, VEGFA
MOL033	Chrysoeriol	NOS2, PTGS1, ESR1, AR, PPARG, PTGS2, ESR2, MAPK14, GSK3B, CDK2, PIK3CG, PIM1, ABCG2, AKR1B1, CA3, CA9, CYP1A1, CYP1B1, CREB1, DAPK1, EP300, IL2, RPS6KA3, ALOX5, ABCB1, ABCC1, TTR, VEGFA
MOL039	8-Isopentenyl-kaempferol	ESR2, ESR1, NOS2, AR, PPARG, PTGS2, GSK3B, CDK2, PIM1, PTGS1, F7, MAPK14, KCNMA1, KCNH2, ABCG2, ALDH2, CYP1B1, DAPK1, RPS6KA3, ALOX5, ABCB1, ABCC1, RELA, XDH
MOL067	Yinyanghuo C	NOS2, ESR1, AR, PPARG, PTGS2, ESR2, MAPK14, GSK3B, CDK2, PIM1, ABCG2, AKR1B1, CYP1A1, CYP1B1, CREB1, DAPK1, EP300, IL2, RPS6KA3, ABCB1, ABCC1, TTR, VEGFA, XDH
MOL069	Yinyanghuo E	ESR1, ASIC3, NOS2, AR, PPARG, PTGS2, ESR2, MAPK14, GSK3B, CDK2, PIM1, ABCG2, AKR1B1, CYP1B1, CREB1, DAPK1, IL2, ABCB1, ABCC1, TTR, VEGFA, XDH
MOL074	8-(3-methylbut-2-enyl)-2-phenyl-chromone	NOS2, PTGS1, ESR1, AR, PPARG, PTGS2, NOS3, ADRB2, ESR2, MAPK14, GSK3B, CDK2, PIM1, ABCG2, CYSLTR1, CYP1A1, CYP1B1, CREB1, ABCB1, PRKDC
MOL012	(2S)-7-hydroxy-2-(4-hydroxyphenyl)-4-chromanone	MMP9, JUN, ABCG2, ESR1, ESR2, LDHA, MALT1, VEGFA, NOS2, PTGS1, AR, PPARG, PTGS2, ADRB2, MAPK14, GSK3B, CDK2, PIK3CG, PIM1
MOL065	Yinyanghuo A	ASIC3, ESR1, AR, PTGS2, PIM1, CYP1B1, CREB1, DAPK1, ABCB1, ABCC1, TTR, VEGFA, XDH
MOL106	Icariin	PDE5A, APEX1, ABCG2, AKR1B1, CA12, CYP1B1, IL2, RPS6KA3, LGALS3, TYR, XDH
MOL004	24-epicampesterol	ADCY1, DHCR7, PPARG, ESR1, AR, PGR, PTGS1, CA1, CA2, CYP19A1, CYP24A1, ESR2, FGF1, ABCC4, RORC, SREBF2, VDR, GC
MOL010	Poriferast-5-en-3beta-ol	ADCY1, DHCR7, PPARG, ESR1, AR, PGR, NR3C1, CA1, CA2, CYP19A1, CYP24A1, ESR2, ABCC4, RORC, SREBF2, VDR, GC
MOL042	Sitosterol	GSK3B, ESR1, AR, PGR, NR3C1, AR, CA1, CA2, CYP19A1, CYP24A1, ESR1, ESR2, ABCC4, RORC, SREBF2, VDR, GC
MOL008	Linoleyl acetate	PTGS1, PPARG, PTGS2, NOS3, ESR1, CNR1, CNR2, ENPP2, CES1, FABP4, PRKCA, ALOX5, PLA2G4A, PDCD4, S1PR2, STS, TLR2, TRPV1
MOL063	C-Homoerythrinan, 1,6-didehydro 3,15,16-trimethoxy-, (3.beta.)-	ADCY1, KCNA5, PTGS1, KCNH2, ESR1, AR, PTGS2, ADRA2A, HTR1A, HRH1, ADRA2B, ADRB2, CHRNA2, DRD2, ESR2, GSK3B, CHRNA7, CHRNA5
MOL077	Anhydroicaritin-3-O-alpha-L-rhamnoside	TF, VDR, PLK2, ABCG2, AKR1B1, CYP1B1, IL2, RPS6KA3, LGALS3, PDE5A, TYR, XDH

Supplementary Table 2. Active compounds for each potential target.

Potential targets	MOL-ID (Active compounds)
ESR1	MOL004, MOL008, MOL010, MOL012, MOL033, MOL039, MOL042, MOL044, MOL056, MOL063, MOL065, MOL067, MOL069, MOL074,
AR	MOL004, MOL008, MOL010, MOL012, MOL033, MOL039, MOL042, MOL044, MOL056, MOL063, MOL065, MOL067, MOL069, MOL074
ESR2	MOL004, MOL010, MOL012, MOL033, MOL039, MOL042, MOL044, MOL056, MOL063, MOL067, MOL069, MOL074
PPARG	MOL004, MOL008, MOL010, MOL012, MOL033, MOL039, MOL044, MOL056, MOL067, MOL069, MOL074
PTGS2	MOL008, MOL012, MOL033, MOL039, MOL044, MOL056, MOL063, MOL065, MOL067, MOL069, MOL074
ABCG2	MOL012, MOL033, MOL039, MOL044, MOL056, MOL067, MOL069, MOL074, MOL077, MOL106
GSK3B	MOL012, MOL033, MOL039, MOL042, MOL044, MOL056, MOL063, MOL067, MOL069, MOL074
CYP1B1	MOL033, MOL039, MOL044, MOL056, MOL065, MOL067, MOL069, MOL074, MOL077, MOL106
PTGS1	MOL004, MOL008, MOL012, MOL033, MOL039, MOL044, MOL056, MOL063, MOL074
PIM1	MOL012, MOL033, MOL039, MOL044, MOL056, MOL065, MOL067, MOL069, MOL074
NOS2	MOL012, MOL033, MOL039, MOL044, MOL056, MOL067, MOL069, MOL074
MAPK14	MOL012, MOL033, MOL039, MOL044, MOL056, MOL067, MOL069, MOL074
CDK2	MOL012, MOL033, MOL039, MOL044, MOL056, MOL067, MOL069, MOL074
XDH	MOL039, MOL044, MOL056, MOL065, MOL067, MOL069, MOL077, MOL106
CREB1	MOL033, MOL044, MOL056, MOL065, MOL067, MOL069, MOL074
DAPK1	MOL033, MOL039, MOL044, MOL056, MOL065, MOL067, MOL069
IL2	MOL033, MOL044, MOL056, MOL067, MOL069, MOL077, MOL106
RPS6KA3	MOL033, MOL039, MOL044, MOL056, MOL067, MOL077, MOL0106
ABCB1	MOL033, MOL039, MOL056, MOL065, MOL067, MOL069, MOL074
VEGFA	MOL012, MOL033, MOL044, MOL065, MOL067, MOL069
AKR1B1	MOL033, MOL044, MOL067, MOL069, MOL077, MOL0106
ABCC1	MOL033, MOL039, MOL056, MOL065, MOL067, MOL069
ALOX5	MOL008, MOL033, MOL039, MOL044, MOL056,
PGR	MOL004, MOL010, MOL042, MOL044
CA2	MOL004, MOL010, MOL042, MOL044
CYP19A1	MOL004, MOL010, MOL042, MOL056
VDR	MOL004, MOL010, MOL042, MOL077
NOS3	MOL008, MOL044, MOL056, MOL074
ADRB2	MOL012, MOL056, MOL063, MOL074
TTR	MOL033, MOL065, MOL067, MOL069
KCNH2	MOL039, MOL044, MOL056, MOL063
ADCY1	MOL004, MOL010, MOL063
CA1	MOL004, MOL010, MOL042
CYP24A1	MOL004, MOL010, MOL042
ABCC4	MOL004, MOL010, MOL042
RORC	MOL004, MOL010, MOL042
SREBF2	MOL004, MOL010, MOL042
GC	MOL004, MOL010, MOL042
PIK3CG	MOL012, MOL033, MOL044
CYP1A1	MOL033, MOL067, MOL074
PDE5A	MOL056, MOL077, MOL106
F7	MOL039, MOL044, MOL056
DHCR7	MOL004, MOL010
NR3C1	MOL010, MOL042
CA3	MOL033, MOL044
EP300	MOL0393, MOL067
KCNMA1	MOL039, MOL056
ALDH2	MOL039, MOL044
RELA	MOL039, MOL056
APEX1	MOL044, MOL0106
ASIC3	MOL065, MOL069
LGALS3	MOL077, MOL0106

TYR	MOL077, MOL0106
FGF1	MOL004
CNR1	MOL008
CNR2	MOL008
ENPP2	MOL008
CES1	MOL008
FABP4	MOL008
PRKCA	MOL008
PLA2G4A	MOL008
PDCD4	MOL008
S1PR2	MOL008
STS	MOL008
TLR2	MOL008
TRPV1	MOL008
MMP9	MOL012
JUN	MOL012
LDHA	MOL012
MALT1	MOL012
CA9	MOL033
CYP1A2	MOL044
RXRB	MOL056
HRH1	MOL063
NCOA1	MOL056
NQO1	MOL056
KCNA5	MOL063
ADRA2A	MOL063
HTR1A	MOL063
ADRA2B	MOL063
CHRNA2	MOL063
DRD2	MOL063
CHRNA7	MOL063
CHRNA5	MOL063
CYSLTR1	MOL074
CA12	MOL0106
PRKDC	MOL074
TF	MOL077
PLK2	MOL077
